# **ItaCORMs**: conjugation with a CO-releasing unit greatly enhances the anti-inflammatory activity of itaconates†

**DOI:** 10.1039/d1md00163a

**Published:** 2021-10-15

**Authors:** Bernhard M. Krause, Britta Bauer, Jörg-Martin Neudörfl, Thomas Wieder, Hans-Günther Schmalz

**Affiliations:** University of Cologne, Department of Chemistry Greinstr. 4 5939 Köln Germany schmalz@uni-koeln.de; University Medical Center Tübingen, Department of Dermatology Liebermeisterstr. 25 72076 Tübingen Germany; Physiologisches Institut, Abteilung für Vegetative und Klinische Physiologie, Eberhard-Karls-Universität Tübingen Wilhelmstr. 56 72074 Tübingen Germany

## Abstract

Endogenous itaconate as well as the gasotransmitter CO have recently been described as powerful anti-inflammatory and immunomodulating agents. However, each of the two agents comes along with a major drawback: Whereas itaconates only exert beneficial effects at high concentrations above 100 μM, the uncontrolled application of CO has strong toxic effects. To solve these problems, we designed hybrid prodrugs, *i.e.* itaconates that are conjugated with an esterase-triggered CO-releasing acyloxycyclohexadiene–Fe(CO)_3_ unit (**ItaCORMs**). Here, we describe the synthesis of different **ItaCORMs** and demonstrate their anti-inflammatory potency in cellular assays of primary murine immune cells in the low μmolar range (<10 μM). Thus, **ItaCORMs** represent a promising new class of hybrid compounds with high clinical potential as anti-inflammatory agents.

## Abstract 2

New powerful anti-inflammatory agents (prodrugs) were developed which act by esterase-triggered, simultaneous release of itaconate and the gasotransmitter carbon monoxide.

## Graphical abstract

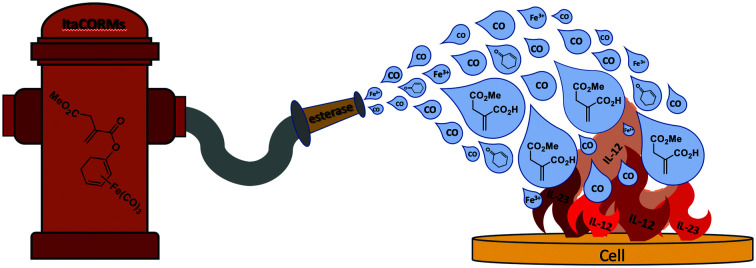

## Introduction

Dimethyl fumarate (**DMF**; 1) represents one of the structurally simplest active pharmaceutical ingredients. Since its accidental discovery, it has become one of the most frequently used immunomodulatory and anti-inflammatory agents in the clinics, especially for the treatment of psoriasis and multiple sclerosis.^1^ As an electrophilic molecule **DMF** was shown to covalently modify cysteine residues, thereby inactivating proteins such as the glycolytic enzyme glyceraldehyde 3-phosphate dehydrogenase (GAPDH) and causing down-regulation of aerobic glycolysis. Another relevant target of **DMF** is the Kelch-like ECH-associated protein 1 (KEAP1) which is involved in inflammatory processes by suppressing the (erythroid-derived 2)-related factor 2 (Nrf2).^2^

The endogenous metabolite itaconic acid (**IA**; 2) has recently been identified as an even stronger anti-inflammatory and immunoregulatory agent^3^ also modulating macrophage function by activating Nrf2 *via* alkylation of cysteine residues of KEAP1. Moreover, the cell-permeable derivative 4-octyl itaconate (**4-OI**; 3) proved to be a strong protective agent against lipopolysaccharide (LPS) induced lethality of human peripheral blood mononuclear cells (PBMCs) *in vivo* by reducing cytokine production.^4^ In another study, the extensive metabolic rewiring of inflammatory macrophages, including regulation of the IκBζ–ATF3 inflammatory axis, was shown to be linked to the induction of electrophilic stress caused by **IA** and even stronger by its membrane-permeable derivative dimethyl itaconate (**DMI**; 4) by selectively inhibiting a subset of cytokines, including IL-6 and IL-12.^5^ These effects were confirmed in subsequent studies, and the promising potential of itaconate-derived compounds as therapeutic agents is reflected by intense ongoing research activities in leading laboratories.^6^

**Scheme 1:**
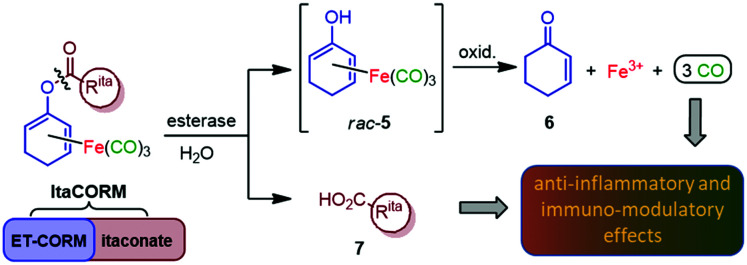
Design of anti-inflammatory compounds (**ItaCORMs**) acting by intracellular esterase-triggered release of both carbon monoxide (CO) and an itaconate (7).

Another most simple anti-inflammatory, immunomodulating and cytoprotective molecule is carbon monoxide (CO), a so-called gasotransmitter which, like NO, displays highly beneficial effects at very low concentrations.^7^ To avoid the risks and toxicity associated with administration of CO by inhalation, the development of methods for the targeted delivery of CO, particularly through the use of CO-releasing molecules (CORMs), has become a fascinating field of drug discovery.^8^ While a variety of CORMs have been reported in the literature, most of them do not meet the requirements for pharmaceutical application. A promising concept to meet the challenge of controlled (intracellular) CO release is the use of enzyme-triggered CORMs (ET-CORMs).^9^ In this context, we introduced acyloxydiene–Fe(CO)_3_ complexes activated by enzyme-induced hydrolysis of the ester moiety.^10^ In contrast to the stable ester prodrugs, the resulting dienol–Fe(CO)_3_ complexes, such as *rac*-5, undergo rapid oxidation under physiological conditions, leading to the release of up to three molecules of CO causing the expected pronounced biological effects (Scheme 1). Noteworthy, simultaneously formed cyclohexenone (6) and ferric ions (Fe^3+^) were found not to affect cell viability at the relevant concentrations.^11^ Another by-product of the enzymatic ester cleavage of ET-CORMs is a carboxylic acid corresponding to the acyloxy unit. While first studies on our esterase-triggered system were conducted only with biologically non-active acid residues, such as acetate or homologous alkanoic acids, we were aware that the ET-CORM concept ideally allows a bioactive acid to be concomitantly released together with CO which could act synergistically. Hence, methyl fumarate-derived ET-CORMs (FumET-CORMs) were presented as one of the first bifunctional CORMs which exhibited a substantially improved anti-inflammatory effect as compared to the clinically used drug **DMF** (1).^12^ In a somewhat related fashion, (untriggered) hybrid CORMs, derived from fumarates and Co, Mn or Ru carbonyl complex units have been reported by Motterlini and coworkers.^13^

Inspired by the promising properties of the FumET-CORMs and taking into account the significantly higher bioactivity of **DMI** (4)^3–5^ compared to **DMF** (1), we now envisioned to synthesize and investigate itaconate-derived ET-CORMs (**ItaCORMs**) which we expected to exhibit even more powerful anti-inflammatory effects (Scheme 1).

## Results

### Synthesis of **ItaCORMs**

**Scheme 2:**
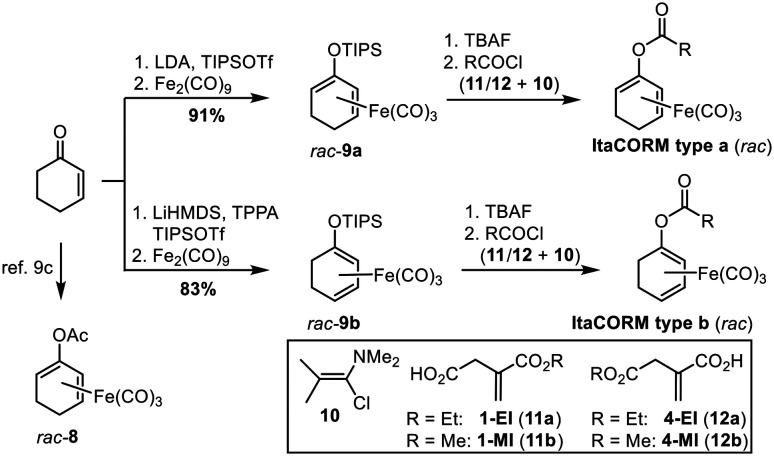
Synthesis of various itaconate-derived acyloxycyclohexadiene–Fe(CO)_3_ complexes as potential CO-releasing molecules (**ItaCORMs**). LDA = lithium diisopropylamide; TIPSOTf = triisopropylsilyl trifluoromethanesulfonate; TPPA = tripyrolidinophosphoric acid triamide; TBAF = tetrabutylammonium fluoride.

According to our previously established procedure, **ItaCORMs** were prepared from the cyclohexenone-derived silyl dienol ethers *rac*-9a or *rac*-9b, respectively (Scheme 2).^9*c*^ For this purpose, the silyl group of *rac*-9a/b was first cleaved off with TBAF in THF and the resulting iron-complexed dienolate alkoxides were reacted with the respective itaconate-derived acid chlorides prepared *in situ* using Ghosez's reagent (10).^14^ The required mono-alkyl itaconates were either commercially obtained (12a/12b) or synthesized (11a/11b) from itaconic acid following a modified literature protocol.^15^ These compounds were also employed as controls in the biological investigations (*vide infra*). As an additional reference substance, the known acetic acid-derived ET-CORM *rac*-8 was prepared as previously described.^10^

According to the general protocol (Scheme 2), a set of eight (racemic) **ItaCORMs** were prepared in good to excellent yield (Fig. 2). Noteworthy, initial attempts to perform the esterification of the intermediate dienolate–Fe(CO)_3_ complexes under Steglich-type conditions (previously used to prepare fumarate-derived CORMs)^12^ completely failed in the itaconate series.

The prepared (racemic) **ItaCORMs** were all characterized by the common spectroscopic methods and proved to be stable and easy to handle compounds, which can be stored for longer periods of time in the fridge without decomposition. The structures of the methyl ester derivatives **ItaCORM**3b and **ItaCORM**4b were additionally confirmed by single crystal X-ray analysis. (Fig. 3).

### *In vitro* CO-release

To probe the proposed ability of the **ItaCORMs** to undergo enzyme-triggered cleavage/oxidation under concomitant carbon monoxide (CO) release (Scheme 1) samples of the complexes were treated with pig liver esterase (PLE) in a DMSO/phosphate buffer mixture, and the CO release was monitored over a period of 100 h by means of headspace gas chromatography. While virtually no CO (≤0.05 equiv.) was released in the absence of PLE (control) we found that significant amounts of CO were liberated from the **ItaCORMs** in the presence of PLE (Fig. 4). Interestingly, but not completely unexpected,^10,12^ the **ItaCORMs** of type a, with the acyloxy substituent at the “inner” position of the cyclohexadiene–Fe(CO)_3_ moiety (Fig. 4A), showed a considerably slower CO release in comparison to the “outer” series (Fig. 4B). We also found that **ItaCORMs**4a and 4b, derived from 4-methyl itaconate (12b) displayed a stronger tendency to release CO than the isomers derived from 1-methyl itaconate (11b). In this particular assay, the “best” CO-releasing molecule of the investigated compounds proved to be **ItaCORM**4b, which released three full equivalents of CO within 17 hours (Fig. 4B). We would like to emphasize, however, that these *in vitro*-measured CO-release kinetics reflect the sum of several steps including of the ester cleavage and the oxidation of the primary dienol–Fe(CO)_3_ complexes of type *rac*-5 (Scheme 1).

### Biological studies

To investigate the expected anti-inflammatory activity of **ItaCORMs** we employed an established *in vitro* model for the analysis of inflammatory responses utilizing bone marrow-derived dendritic cells (BMDCs) stimulated by lipopolysaccharide (LPS).^12,16^ At first, we determined the cytotoxicity of **ItaCORMs**1a/b and 2a/2b by means of the XTT assay,^12^ and an assay based on the release of lactate dehydrogenase (LDH).^17^ While these compounds showed no substantial toxicity up to a concentration of 15 μM, a rapid decrease of the cell viability was observed at concentrations of ≥25 μM for all **ItaCORMs** with the exception of **ItaCORM**1a (see Fig. SI-9†). Therefore, all subsequent experiments were performed using non-toxic concentrations ≤15 μM.

**Fig. 1:**
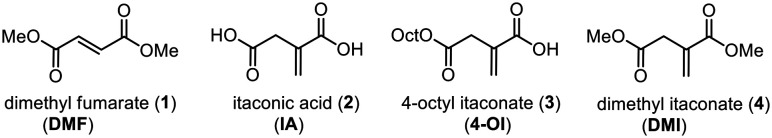
Dimethyl fumarate and itaconic acid derivatives displaying pronounced anti-inflammatory effects.

As previously shown, the envisaged induction of an anti-inflammatory type II phenotype of dendritic cells importantly depends both on the regulation of HO-1 and STAT1 signaling pathways and on the associated inhibition of inflammatory cytokines IL-23 and IL12-p70.^12^ Hence, we next analyzed the effects of **ItaCORMs** on these signaling mechanisms (Fig. 5). And indeed, HO-1 was strongly upregulated upon treatment of the cells with **ItaCORMs**1b, 2a and 2b, in contrast to STAT1, which remained more or less unchanged. Noteworthy, the effects of these dual-active compounds proved to be much stronger than those of the control substances **1-EI** (11a), **4-EI** (12a) and *rac*-8, respectively, which only release either itaconic acid or CO (Fig. 1).

**Fig. 2:**
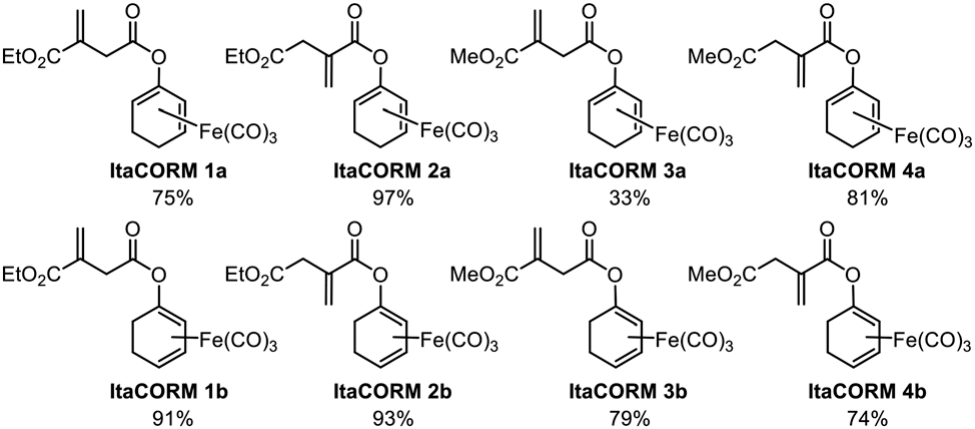
Itaconate-derived CO-releasing molecules (**ItaCORMs**) synthesized according to Scheme 2.

**Fig. 3:**
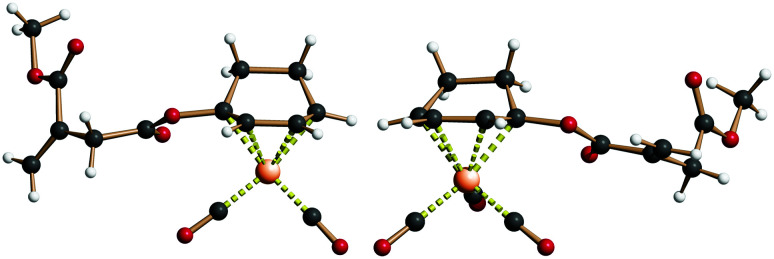
Structure of **ItaCORM**3b (left) and **ItaCORM**4b (right) in the crystalline state.

**Fig. 4:**
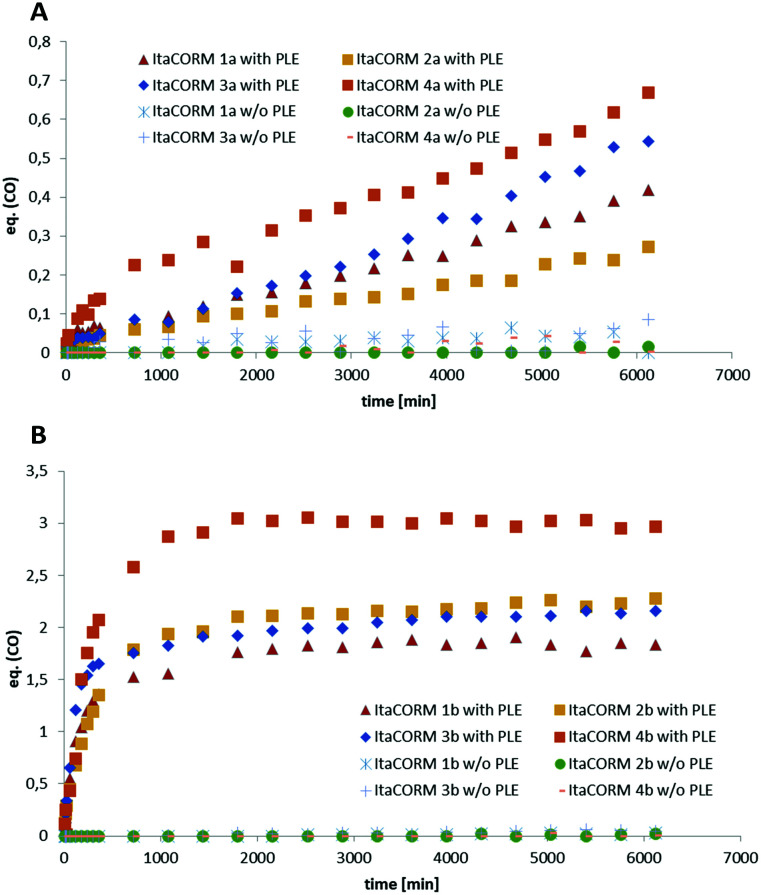
Quantification of the PLE-triggered CO release properties of **ItaCORMs**. A: **ItaCORMs** of type **a**. B: **ItaCORMs** of type **b**. Each complex (36 μmol) was dissolved in 0.2 mL of DMSO and 1.0 mL of phosphate buffer (pH 7.4) before 15 mg of PLE (≥24 units per mg) were added. The resulting mixture was stirred at 37 °C and the released CO was measured *via* headspace gas chromatography. Samples of each **ItaCORM** in the absence of PLE were analyzed in *via* the same method as a control.

**Fig. 5:**
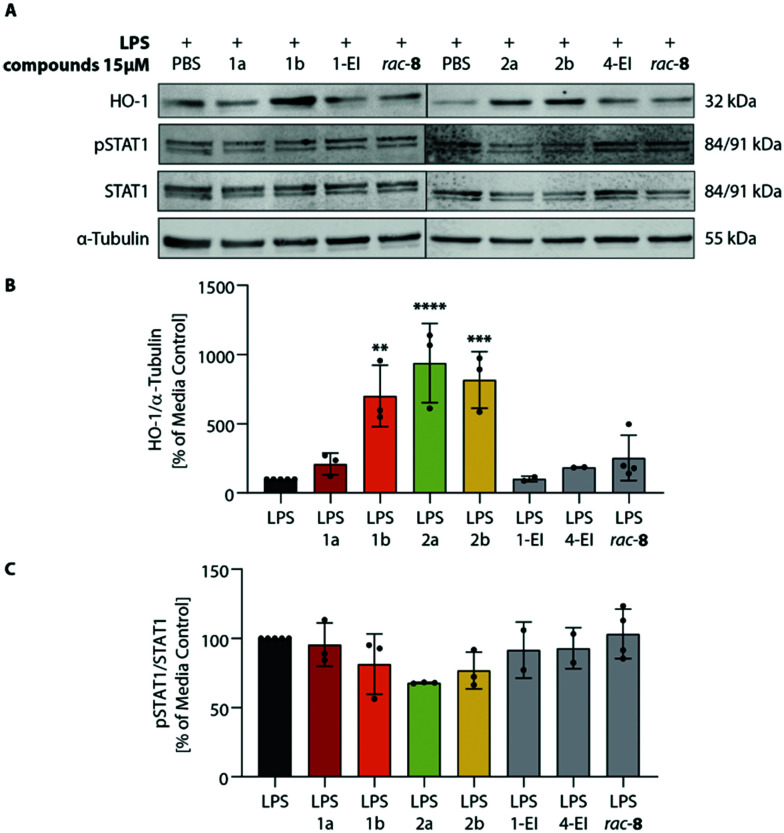
Modulation of HO-1 and STAT1 signaling pathways by **ItaCORMs**. BMDCs were stimulated with 1 μg ml^−1^ LPS and co-incubated with PBS, in the presence of 15 μM of **ItaCORMs**, or 1-**EI**, 4-**EI** and *rac*-8 as control substances. After 2 h, cell lysates were harvested and analyzed by Western blot using HO-1, STAT1, pSTAT1 and α-tubulin antibodies. (A) Representative Western blots. As loading control α-tubulin was used. (B and C) The video-densiometric quantification of the HO-1/α-tubulin (B) and pSTAT1/STAT1 (C) ratios are shown in percent of the LPS-treated control group. Values are means ± SEM (*n* = 2–5); **, *p* ≤ 0.01; ****, *p* ≤ 0.0001 (ANOVA with Tukey *post hoc* test).

**Fig. 6:**
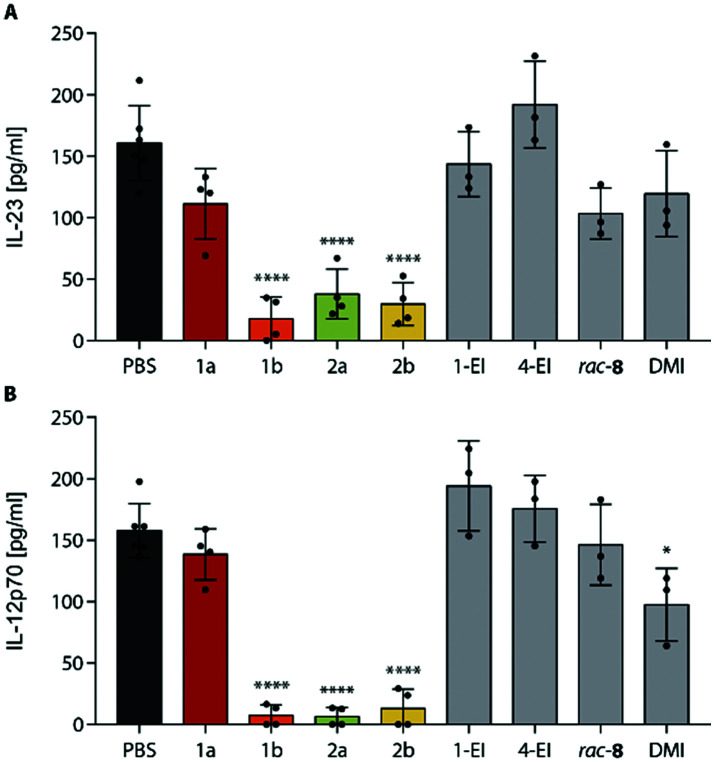
Strong inhibition of inflammatory cytokines by **ItaCORMs**. Concentrations of IL-23 (A) and IL-12p70 (B) were determined in the supernatant of BMDCs stimulated with 1 μg ml^−1^ LPS and co-incubated with PBS, 15 μM **ItaCORMs**, or 1-**EI**, 4-**EI**, *rac*-8, and **DMI** as control substances for 24 h. Values are means ± SEM (*n* = 3–6); *, *p* ≤ 0.05; ****, *p* ≤ 0.0001 (ANOVA with Tukey *post hoc* test).

**Fig. 7:**
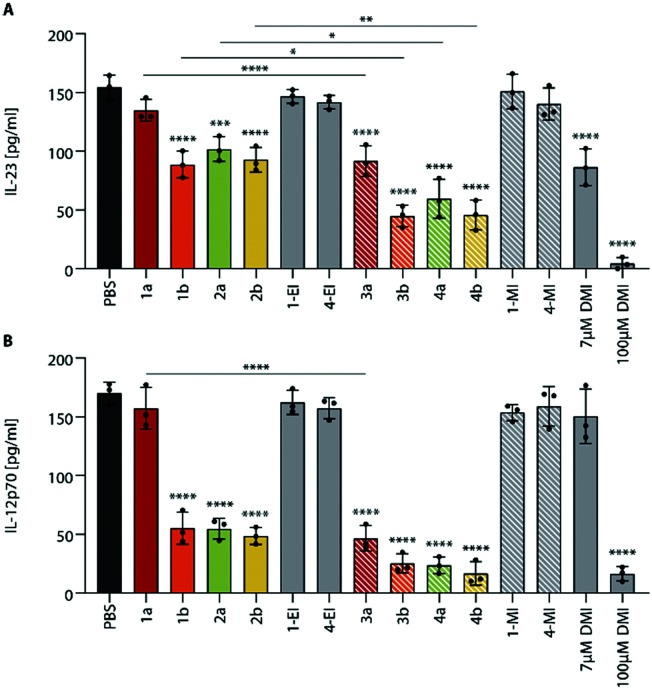
Comparison of ethyl itaconate (fully colored bars) and methyl itaconate (dashed bars) derived **ItaCORMs**. BMDCs were stimulated with 1 μg ml^−1^ LPS and co-incubated with PBS, ethyl-**ItaCORMs**1a, 1b, 2a, 2b, methyl-**ItaCORMs**3a, 3b, 4a, 4b or control compounds (7 μM). After 24 h of incubation, cell culture supernatants were collected and analyzed for IL-23 (A) and IL-12p70 (B) cytokine levels. Values are means ± SEM (*n* = 3); *, *p* ≤ 0.05; **, *p* ≤ 0.01; ***, *p* ≤ 0.001; ****, *p* ≤ 0.0001 (ANOVA with Tukey *post hoc* test).

**Fig. 8:**
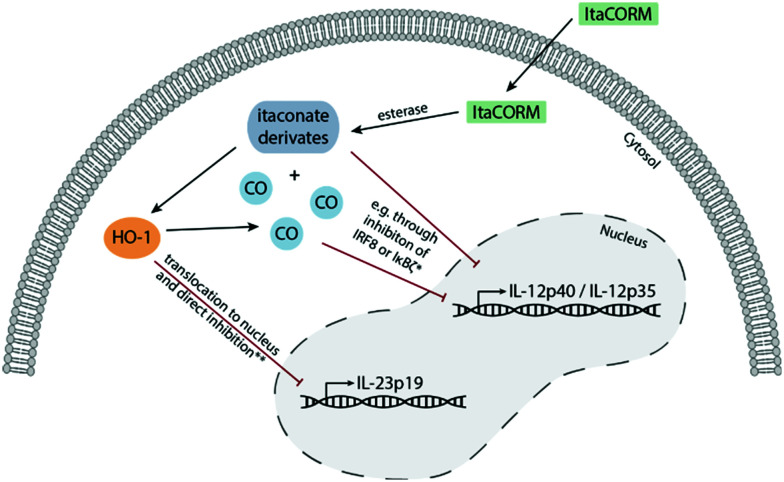
Possible mechanisms of the anti-inflammatory activity of **ItaCORMs**. After cleavage of **ItaCORMs** by esterases, itaconate and CO are released into the cytoplasm of the cell. This results in the induction of heme oxygenase (HO-1), which, after translocation into the nucleus, is responsible for the inhibition of IL-23 expression. Furthermore, itaconate and CO cause decreased levels of Il-12, possibly due to inhibition of transcription factors, *e.g.* the interferon regulatory factor 8 (IRF8) or IκBζ as the inhibitor of NF-κBζ.

As another activity, we also observed a strong downregulation of inflammatory cytokines IL-23 and IL-12p70 upon treatment of LPS-stimulated BMDCs with **ItaCORMs** (Fig. 6). While **ItaCORM**1a again showed only minor effects (as in the HO-1 induction), **ItaCORMs**1b, 2a, and 2b caused a pronounced reduction of cytokine levels. Again, the latter compounds proved to be much more potent than any of the controls applied at the same concentration. Noteworthy, dimethyl itaconate (**DMI**), used as a highly active benchmark compound in the recent literature,^5^ showed only a minor effect at these concentrations.

Since the itaconate esters derived from different alcohols may differ in their biological activity,^5^ we also investigated the methyl itaconate-derived **ItaCORMs**3a, b and 4a, b in comparison to the ethyl esters (**ItaCORMs**1a, 1b, 2a, 2b) with regard to their ability to inhibit the expression of inflammatory cytokines (Fig. 7).

We found, that at the low concentration of 7 μM the methyl esters caused an even stronger decrease of IL-23 and IL-12p70 than the initially studied ethyl esters. As Fig. 7 reveals, the relative activity within the two series proved to be similar. Remarkably, an almost complete suppression of IL-23 was observed upon treatment of BMDCs with **ItaCORMs**3b, 4a or 4b at a concentration of 15 μM (ESI† Fig. S10). At this concentration, IL-12p70 totally disappeared in the presence of all **ItaCORMs** investigated except for 1a and 3a, the latter being also highly active at this concentration. Fig. 7 also indicates again the superb anti-inflammatory efficacy of the **ItaCORMs** as compared to 7 μM dimethyl itaconate (**DMI**), which displayed comparable effects only at much higher concentrations (100 μM).

## Discussion

While gaseous CO is highly toxic upon inhalation it has beneficial cellular effects at low concentrations. Physiologically, CO is produced by HO-1, the enzyme that catalyzes the rate-limiting step in heme degradation. CO has been shown to mediate the beneficial effects of HO-1, including protection against oxidative stress, modulation of the inflammatory response and regulation of angiogenesis.^18^ Our data that **ItaCORMs** induce HO-1 at low concentrations therefore point to a positive feed-forward mechanism that leads to sustained activation of the HO-1/CO axis. Furthermore, **ItaCORMs** as presented in this study combine esterase-triggered (intracellular) co-release of CO and an anti-inflammatory itaconate. The results indicate that the two agents then synergistically activate two pathways: one leading to HO-1-dependent downregulation of IL-23 and the other one resulting in repression of IL-12p70, presumably *via* inhibition of IRF8 or IκBζ (Fig. 8). Together, this promotes an anti-inflammatory type II immune response.^19^ Regarding this, **ItaCORMs** proved to be by far more effective than the single treatment with an itaconate, CO alone or other related CORMs. Particularly, the concentrations of itaconates required to induce anti-inflammatory cellular effects in the absence of a CO source are in the range of 100 μM.^4,5^ This is about 10 times higher than the effective concentration of **ItaCORMs** described in the study.

## Experimental

### General procedure for the synthesis of **ItaCORMs**

(Reactions were run on a 127–520 μmol scale under argon.) In a Schlenk tube, a 0.3 M solution of the respective itaconic monoester (11 or 12) (1.4–1.5 eq.) in CH_2_Cl_2_ was cooled to 0 °C. Ghosez's reagent (1.5–1.7 eq.) was added and the mixture was stirred for 1 h (M1). In a separate Schlenk tube, a 0.3–0.5 M solution of complex *rac*-9a or *rac*-9b (1.0 eq.) in CH_2_Cl_2_ was cooled to 0 °C and TBAF (1 M in THF, 1.0 eq.) was added dropwise under stirring (M2). After 10 min solution M2 was transferred dropwise into M1 at 0 °C. The resulting solution was stirred at 0 °C for 10 min and then for 16–21 h at room temperature. The solvent was removed under reduced pressure and the crude product was purified by column chromatography on SiO_2_ (Cyhex/EtOAc; 20/1 to 5/1) to give the respective **ItaCORM**. For details, including substance data, PLE-triggered CO-release experiments and biochemical investigations, see the ESI.†

### Mice

Wildtype C57BL/6J mice were maintained and bred under specific pathogen-free conditions at the premises of the Eberhard Karls University Tübingen. Mouse handling and isolation of cells from bone marrow were approved by the “Einrichtung für Tierschutz, Tierärztlichen Dienst und Labortierkunde” and the “Regierungspräsidium Tübingen” and were performed with female and male animals aged 8 weeks and older. All animal procedures were performed in accordance with the Guidelines for Care and Use of Laboratory Animals of the University of Tübingen Germany. All animal experiments were approved by the local authorities (Regierungspräsidium Tübingen, Germany).

### Isolation of BMDCs

Bone marrow derived dendritic cells were isolated as previously described.^20^ In brief, bone marrow cells were obtained from the femurs and tibias of mice. Differentiation of cells into BMDCs was performed by culture in DMEM media supplemented with 10% fetal calf serum, 1% non-essential amino acids, 1% sodium pyruvate, 1% HEPES, 1% antibiotics, 25 μM β-mercaptoethanol and 20 ng ml^−1^ recombinant granulocyte-macrophage colony-stimulating factor (GM-CSF). Refreshment with new cell culture medium was performed on days 3 and 6. After 7 days, harvest of immature BMDCs was performed by careful collection of non-adherent cells.

### Western blot analysis

BMDCs were treated as indicated in the figure description and protein expression was analyzed as previously described.^12^ Briefly, cell lysates were separated by SDS-PAGE and blotted onto polyvinylidene difluoride transfer membranes. Protein detection was performed by incubation with HO-1, STAT1, pSTAT1 and α-tubulin antibodies and using the Odyssey SA Infrared Imaging System.

### Cytokine analysis

BMDCs were stimulated with LPS and co-incubated with the respective **ItaCORMs** or control substances as indicated in the figure legend. After 24 h, supernatants of each culture condition were collected and assayed for IL-12p70 and IL-23 levels. ELISA was performed using DuoSet ELISA Mouse IL-12p70, DuoSet ELISA Mouse IL-23 and DuoSet Ancillary Reagent Kit 2 (R&D Systems).

## Conclusions

We have synthesized and chemically characterized a new class of acyloxydiene–Fe(CO)_3_ complexes which upon esterase-triggered cleavage disintegrate to concomitantly release carbon monoxide (CO) and an itaconate as two important anti-inflammatory and immunomodulating agents. Some of these compounds, especially the 4-methyl itaconate-derived **ItaCORMs**4a and 4b were then shown in cellular assays to induce HO-1 and to strongly inhibit the expression of inflammatory cytokines IL-23 and IL-12p70 at low micromolar concentration. **ItaCORMs**4a and 4b therefore represent particularly promising anti-inflammatory agents which may deserve further clinical evaluation.

## Author contributions

T. W. and H.-G. S. initiated research; B. M. K. performed chemical syntheses and CO-release studies; B. B. performed biochemical investigations; J.-M. N. performed X-ray crystal structure analyses; B. M. K., B. B., T. W. and H.-G. S. wrote the manuscript.

## Conflicts of interest

There are no conflicts to declare.

## Supplementary Material

MD-012-D1MD00163A-s001

MD-012-D1MD00163A-s002

## References

[cit1] Blair H. A. (2019). Drugs.

[cit2] Kornberg M. D., Bhargava P., Kim P. M., Putluri V., Snowman A. M., Putluri N., Calabresi P. A., Snyder S. H. (2018). Science.

[cit3] Li R., Zhang P., Wang Y., Tao K. (2020). Oxid. Med. Cell. Longevity.

[cit4] Mills E. L., Ryan D. G., Prag H. A., Dikovskaya D., Menon D., Zaslona Z., Jedrychowski M. P., Costa A. S. H., Higgins M., Hams E., Szpyt J., Runtsch M. C., King M. S., McGouran J. F., Fischer R., Kessler B. M., McGettrick A. F., Hughes M. M., Carroll R. G., Booty L. M., Knatko E. V., Meakin P. J., Ashford M. L. J., Modis L. K., Brunori G., Sévin D. C., Fallon P. G., Caldwell S. T., Kunji E. R. S., Chouchani E. T., Frezza C., Dinkova-Kostova A. T., Hartley R. C., Murphy M. P., O'Neill L. A. (2018). Nature.

[cit5] Bambouskova M., Gorvel L., Lampropoulou V., Sergushichev A., Loginicheva E., Johnson K., Korenfeld D., Mathyer M. E., Kim H., Huang L.-H., Duncan D., Bregman H., Keskin A., Santeford A., Apte R. S., Sehgal R., Johnson B., Amarasinghe G. K., Soares M. P., Satoh T., Akira S., Hai T., de Guzman Strong C., Auclair K., Roddy T. P., Biller S. A., Jovanovic M. A., Klechevsky E., Stewart K. M., Randolph G. J., Artyomov M. N. (2018). Nature.

[cit6] Chen M., Sun H., Boot M., Shao L., Chang S.-J., Wang W., Lam T. T., Lara-Tejero M., Rego E. H., Galán J. E. (2020). Science.

[cit7] Motterlini R., Otterbein L. E. (2010). Nat. Rev. Drug Discovery.

[cit8] Romao C. C., Blattler W. A., Seixas J. D., Bernardes G. J. (2012). Chem. Soc. Rev..

[cit9] Ji X., Pan Z., Li C., Kang T., De La Cruz L. K. C., Yang L., Yuan Z., Ke B., Wang B. (2019). J. Med. Chem..

[cit10] Romanski S., Rücker H., Stamellou E., Guttentag M., Neudörfl J.-M., Alberto R., Amslinger S., Yard B., Schmalz H.-G. (2012). Organometallics.

[cit11] Romanski S., Stamellou E., Jaraba J. T., Storz D., Krämer B. K., Hafner M., Amslinger S., Schmalz H.-G., Yard B. A. (2013). Free Radical Biol. Med..

[cit12] Bauer B., Göderz A.-L., Braumüller H., Neudörfl J. M., Röcken M., Wieder T., Schmalz H.-G. (2017). ChemMedChem.

[cit13] Wilson J. L., Kobeissi S. F., Oudir S., Haas B., Michel B., Dubois Randé J.-L., Ollivier A., Martens T., Rivard M., Motterlini R., Foresti R. (2014). Chem. – Eur. J..

[cit14] Munyemana F., George I., Devos A., Colens A., Badarau E., Frisque-Hesbain A.-M., Loudet A., Differding E., Damien J.-M., Rémion J., Van Uytbergen J., Ghosez L. (2016). Tetrahedron.

[cit15] Achiwa K., Chaloner P. A., Parker D. (1981). Organomet. Chem..

[cit16] Madaan A., Verma R., Singh A. T., Jain S. K., Jaggi M. (2014). J. Biol. Methods.

[cit17] Wieder T., Orfanos C. E., Geilen C. C. (1998). J. Biol. Chem..

[cit18] Loboda A., Jozkowicz A., Dulak J. (2015). Vasc. Pharmacol..

[cit19] Ghoreschi K., Brück J., Kellerer C., Deng C., Peng H., Rothfuss O., Hussain R. Z., Gocke A. R., Respa A., Glocova I., Valtcheva N., Alexander E., Feil S., Feil R., Schulze-Osthoff K., Rupec R. A., Lovett-Racke A. E., Dringen R., Racke M. K., Röcken M. (2011). J. Exp. Med..

[cit20] Madaan A., Verma R., Singh A. T., Jain S. K., Jaggi M. (2014). J. Biol. Methods.

