# Supplementary material for: ItaCORMs: conjugation with a CO-releasing unit greatly enhances the anti-inflammatory activity of itaconates
Source: RSC Med Chem. 2021 Oct 15;12(12):2053–9. doi: 10.1039/d1md00163a (PMC8672850; doi:10.1039/d1md00163a)
Supplement: MD-012-D1MD00163A-s001 [file MD-012-D1MD00163A-s001.pdf]

Electronic Supplementary Material (ESI) for RSC Medicinal Chemistry.
This journal is © The Royal Society of Chemistry 2021

Content

1 Chemistry – Experimental Procedures and Data 2

1.1 General Procedures 2

1.2 Synthesis of Itaconate building blocks 4

1.3 Synthesis of Itaconate-linked ET-CORMs (ItaCORMs) 6

1.4 In situ quantification of CO-release 16

1.5 NMR-Spectra 21

1.6 Crystal data and structure refinement 31

2 Biology – Experimental Procedures 33

2.1 Animals 33

2.2 Isolation of primary dendritic cells 33

2.3 Viability 33

2.4 Western blot 34

2.5 Analyses of cytokines 34

3 Biology - Results 34

3.1 Determination of cell viability 34

3.2 ELISA 35

4 References 36

1 Chemistry – Experimental Procedures and Data

1.1 General Procedures

Inert gas

Unless otherwise stated, all reactions were run under an inert atmosphere using *Schlenk* technique and argon 4.6 of the company *Linde* (99.996%, <1 ppm H_2O, <1 ppm O_2). Before being used glassware was evacuated, heated out with a heat gun and then flushed with argon three times.

Solvents and reagents

Unless otherwise stated all chemicals and solvents were purchased from commercial suppliers (*Acros*, *Alfa-Aesar*, *Carbolution*, *Merck*, *Rockwood-Lithium*, *Sigma-Aldrich*, *TCI* and *VWR*) and were used without further purification. Di*iso*propylamine, hexamethyldisalazane and 2-cyclohexenone were distilled and stored under argon atmosphere. The concentration of *n*-butyllithium was determined by titration.[1] Non-absolute solvents were distilled before use. Absolute THF and toluene were obtained by distillation under argon atmosphere over sodium with benzophenone used as indicator. CH_2Cl_2 was dried over $CaCl_2$ and distilled on the day of use. Other solvents were used without further purification unless otherwise noted.

Removal of solvents

Solvents were removed under reduced pressure using a rotary evaporator of the company *Büchi* and a membrane pump. The temperature of the water bath was set to 40 °C. Residual solvents were then removed under oil pump vacuum.

Nuclear magnetic resonance

NMR spectra (^{1}H and ^{13}C) were recorded on the devices Avance II 300 (^{1}H NMR: 300 MHz, ^{13}C NMR: 75 MHz), Avance 400 (^{1}H NMR: 400 MHz, ^{13}C NMR: 100 MHz), Avance III 499 (^{1}H NMR: 500 MHz) and Avance AVIII-HD 500 (^{1}H NMR: 500 MHz, ^{13}C NMR: 125 MHz) of the company *Bruker*. Proton as well as carbon chemical shifts δ are reported in ppm (parts per million) relative to tetramethylsilane. All spectra were measured at room temperature. The multiplicity of signals is described with "s" for singlet, "d" for doublet, "t" for triplet as well as "m" for multiplet and “br” for broad signal. Coupling constants are given in Hertz. The assignment of signals was done using suitable 2D-NMR spectra (COSY, HMQC/HSQC, HMBC, NOESY).

Thin layer chromatography (TLC)

Thin layer chromatography was performed using aluminium sheets coated with silica gel 60 F_{254} from the company *Merck*. The spots were visualised with UV-light (254 nm) and basic $KMnO_4$ solution or ninhydrin solution as staining agents. The eluent mixtures are given in volume fractions for each experiment.

Flash column chromatography

Silica gel 60 (0.035 - 0.07 mm, 60 Å) from the company *Acros* was used as stationary phase for the column chromatography. The silica gel was prepared using the suitable eluent mixture and then compressed using pressure. The eluent mixtures are given in volume fractions for each experiment.

Fourier-Transform-Infrared spectroscopy (FT-IR)

FT-IR spectra were recorded at room temperature on a SpectrumTwo spectrometer from *Perkin-Elmer* with ATR technique. The measured bands are reported in cm^{-1} using the following abbreviations: "w" for weak, "m" for medium, "s" for strong and "vs" for very strong signals.

Gas chromatography with mass spectrometric detector (GC-MS)

The GC-MS spectra were measured on an *Agilent Hewlett Packard* 6890 Series Plus instrument with injector and auto sampler with a HP5973 Series mass selective detector. H_2 was used as carrier gas. A capillary column *Macherey-Nagel* Optima 1 MS (30 mm x 0.25 mm, Ø) was used. Measurements were performed using the temperature programme (50-300MS): 50 °C (2 min), heating rate: 25 °C/min, 300 °C (5 min), 320 °C (5 min).

High resolution Electron-Spray-Ionization mass-spectrometry

HR-ESI-MS spectra were measured on a THERMO Scientific LTQ Orbitrap XL-FTMS Analyser of the company *Thermo Fischer*. Ionization was achieved via electrospray method applying a spray voltage of 3.4 kV. The capillary and tube lens voltage was set to 3.0 V.

Melting point

The uncorrected melting points of solids were measured using a B-545 system of the company *Büchi* with a heating rate of 1 °C/min.

1.2 Synthesis of Itaconate building blocks

1.2.1 Synthesis of diethyl itaconate (DEI, 13)

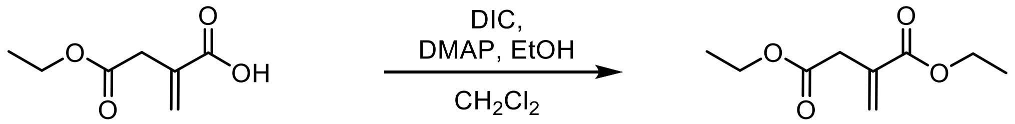

12a **DEI**

A solution of 4-ethyl itaconate (**12a**) (5.00 g, 31.6 mmol, 1.00 eq.) in CH_2Cl_2 (160 mL) was cooled to 0 °C. DIC (6.85 mL, 44.3 mmol, 1.4 eq.), DMAP (1.2 mg, 9.48 mmol, 0.3 eq.) and EtOH (9.23 mL, 158 mmol, 5.00 eq.) were added subsequently. The resulting reaction mixture was stirred at 0 °C for 10 min and then warmed up to room temperature and stirred for 4.5 h. The reaction mixture was filtered over *Celite* with CH_2Cl_2 and concentrated under reduced pressure. The crude product was purified by column chromatography on SiO_2 (Cyhex/EtOAc; 5:1) and the desired product was obtained in a yield of 4.86 g (26.1 mmol, 83%) in form of a colourless oil.

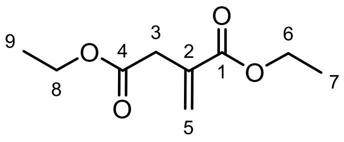

M ($C_9H_{14}O$) 186.21 g/mol.

Yield 4.86 g (26.1 mmol, 83%).

R_f 0.67 (SiO_2, Cyhex/EtOAc; 3:1).

^{1}H NMR (300 MHz, $CDCl_3$): δ [ppm] = 6.32 (s, 1H, H-5a), 5.69 (s, 1H, H-5b), 4.22 (q, *J* = 7.1 Hz, 2H, H-6), 4.16 (q, *J* = 7.1, 2H, H-8), 3.33 (d, *J* = 1.1 Hz, 2H, C-3), 1.30/1.26 (2 x t, *J* = 7.1 Hz, 6H, H-7, H-9).

^{13}C NMR (75 MHz, $CDCl_3$): δ [ppm] = 170.8 (C-4), 166.3 (C-1), 134.3 (C-2), 128.2 (C-5), 61.1 (C-6), 61.0 (C-8), 37.9 (C-3), 2 x 14.3 (C-7, C-9).

FT-IR (ATR): ṽ [cm^{-1}] = 2983 (w), 2940 (w), 2908 (w), 2876 (w), 1737 (s), 1716 (vs), 1640 (m), 1478 (w), 1468 (w), 1447 (w), 1420 (w), 1393 (w), 1369 (m), 1335 (m), 1316 (m), 1300 (m), 1255 (m), 1189 (s), 1144 (vs), 1096 (w), 1030 (s), 947 (m), 887 (w), 861 (m), 818 (m), 786 (w), 762 (w), 741 (w), 648 (w), 629 (w), 573 (w), 557 (w), 515 (w), 432 (w).

The spectroscopic data are in accordance with the literature.[2]

1.2.2 Synthesis of 1-Ethyl itaconate (1-EI, 11a)

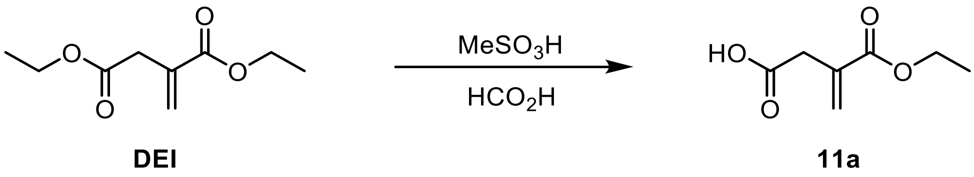

According to a modified procedure by *K. Achiwa*,[3] diethyl itaconate (**DEI**, **13**) (800 mg, 4.30 mmol, 1.00 eq.) was dissolved in formic acid (8 mL) and mixed with $MeSO_3H$ (418 μL, 6.44 mmol, 1.50 eq.). The reaction mixture was heated to 110 °C and stirred for 45 min. After being cooled to room temperature over 1.5 h, the reaction mixture was poured into ice water and extracted with CH_2Cl_2 (4 x 20 mL). The combined organic layers were dried over $MgSO_4$ and the solvent was removed *in vacuo*.

The crude product was purified by *Kugelrohr* distillation and the desired product **11a** was obtained in a yield of 296 mg (1.87 mmol, 44%) in form of a clear colourless oil.

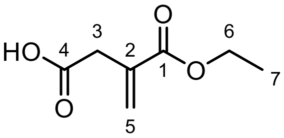

M ($C_7H_{10}O_4$) 158.15 g/mol.

Yield 296 mg (1.87 mmol, 44%).

^{1}H NMR (300 MHz, $CDCl_3$): δ [ppm] = 6.36 (d, J = 1.0 Hz, 1H, H-5a), 5.73 (q, J = 1.2 Hz, 1H, H-5b), 4.24 (q, J = 7.1 Hz, 2H, H-6), 3.39 (d, J = 1.1 Hz, 2H, H-3), 1.30 (t, J = 7.1 Hz, 3H, H-7).

^{13}C NMR (75 MHz, $CDCl_3$): δ [ppm] = 176.9 (C-4), 166.3 (C-1), 133.6 (C-2), 128.9 (C-5), 61.4 (C-6), 37.7 (C-3), 14.2 (C-7).

FT-IR (ATR): ṽ [cm^{-1}] = 3494 (br), 3108 (br), 2985 (w), 2938 (w), 2908 (w), 2874 (w), 2646 (w), 1707 (vs), 1637 (m), 1468 (w), 1446 (w), 1420 (m), 1397 (m), 1372 (m), 1335 (m), 1316 (m), 1300 (m), 1203 (s), 1175 (s), 1150 (s), 1095 (w), 1024 (m), 957 (m), 946 (m), 909 (w), 885 (w), 861 (w), 817 (m), 786 (w), 728 (m), 623 (w), 573 (w), 533 (w), 448 (w).

The spectroscopic data are in accordance with the literature.[2]

1.3 Synthesis of Itaconate-linked ET-CORMs (ItaCORMs)

1.3.1 Synthesis of ItaCORM 1a

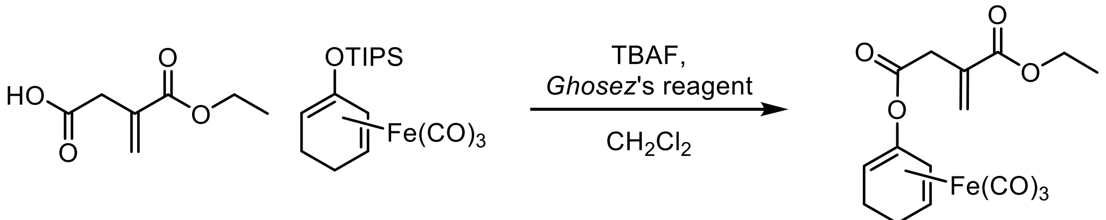

11a *rac*-**9a** **ItaCORM 1a**

In a *Schlenk* tube, 1-ethyl itaconate (**11a**) (28.2 mg, 178 µmol, 1.40 eq.) was dissolved in CH_2Cl_2 (0.6 mL) and cooled to 0 °C. *Ghosez*'s reagent (26.3 µL, 191 µmol, 1.50 eq.) was added and the reaction mixture was stirred for 1.5 h (M1).

In a separate *Schlenk* tube, complex *rac*-**9a** (50 mg, 127 µmol, 1.00 eq.) was dissolved in CH_2Cl_2 (0.6 mL), cooled to 0 °C and TBAF (1 M in THF, 127 µL, 127 µmol, 1.00 eq.) was added dropwise (M2). Upon stirring the mixture for 15 min it was transferred dropwise into M1 at 0 °C. The resulting reaction mixture was stirred at 0 °C for 1 h and then stirred at room temperature for 16 h.

The solvent was removed under reduced pressure and the crude product was purified by column chromatography on SiO_2 (Cyhex/EtOAc; 20:1 to 5:1). The desired product **ItaCORM 1a** was obtained in form of a clear yellow oil in a yield of 36 mg (95.7 µmol, 75%).

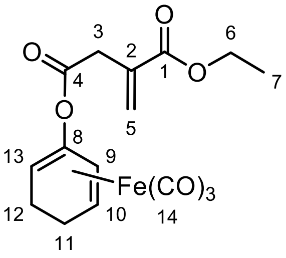

M ($C_{16}H_{16}FeO_7$) 376.14 g/mol.

Yield 36 mg (95.7 µmol, 75%)

R_f 0.79 (SiO_2, Cyhex/EtOAc; 5:1).

^{1}H NMR (500 MHz, $CDCl_3$): δ [ppm] = 6.38 (d, J = 0.8 Hz, 1H, H-5a), 5.77 (d, J = 1.1 Hz, 1H, H-5b), 5.52 (ddd, J = 6.7, 2.1, 0.8 Hz, 1H, H-9), 4.24 (q, J = 7.1 Hz, 2H, H-6), 3.45 (dd, J = 3.7, 1.1 Hz, 2H, H-3), 3.38 (dt, J = 3.8, 2.3 Hz, 1H, H-13), 2.85 (ddd, J = 6.3, 3.5, 2.4 Hz, 1H, H-10), 1.87 – 1.78 (m, 1H, H-12a), 1.79 – 1.70 (m, 1H, H-12b), 1.60 – 1.51 (m, 2H, H-11), 1.31 (t, J = 7.1 Hz, 3H, H-7).

^{13}C NMR (125 MHz, $CDCl_3$): δ [ppm] = 210.8 (C-14), 170.2 (C-4), 166.0 (C-1), 133.3 (C-2), 129.1 (C-5), 128.5 (C-8), 79.8 (C-9), 61.4 (C-6), 59.1 (C-13), 52.2 (C-10), 37.9 (C-3), 24.7 (C-12), 23.6 (C-11), 14.3 (C-7).

HR-MS (ESI) calc.: $[M+Na]^+$ = 399.01377 amu found: 399.01380 amu.

FT-IR (ATR): ṽ [cm^{-1}] = 3007 (w), 2986 (w), 2935 (w), 2903 (w), 2887 (w), 2858 (w), 2045 (vs), 1958 (vs), 1763 (m), 1714 (m), 1639 (w), 1468 (w), 1458 (m), 1430 (w), 1420 (w), 1394 (w), 1372 (w), 1336 (m), 1316 (m), 1255 (w), 1236 (w), 1207 (m), 1169 (s), 1120 (s), 1076 (w), 1025 (m), 996 (w), 958 (w), 925 (w), 900 (w), 882 (w), 861 (w), 839 (w), 818 (w), 791 (w), 757 (w), 725 (w), 678 (w), 618 (m), 608 (s), 571 (s), 507 (m), 460 (w), 416 (w).

1.3.2 Synthesis of ItaCORM 1b

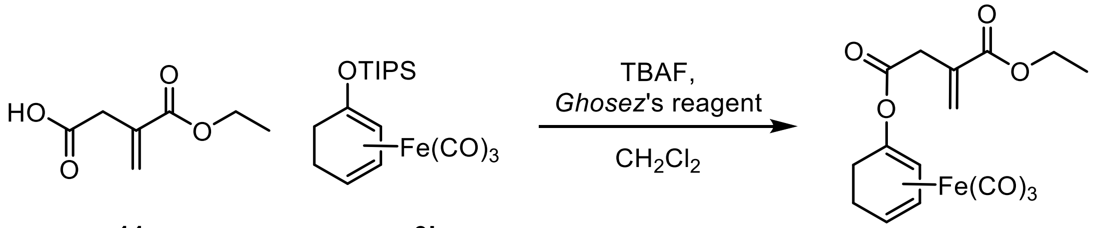

11a *rac*-**9b** **ItaCORM 1b**

In a *Schlenk* tube, 1-ethyl itaconate (**11a**) (84.7 mg, 535 *µ*mol, 1.40 eq.) was dissolved in CH_2Cl_2 (1.3 mL) and cooled to 0 °C. *Ghosez*'s reagent (79.0 *µ*L, 573 *µ*mol, 1.50 eq.) was added and the reaction mixture was stirred for 1.5 h (M1).

In a separate *Schlenk* tube, complex *rac*-**9b** (150 mg, 382 *µ*mol, 1.00 eq.) was dissolved in CH_2Cl_2 (1.3 mL), cooled to 0 °C and TBAF (1 M in THF, 382 *µ*L, 382 *µ*mol, 1.00 eq.) was added dropwise (M2). Upon stirring the mixture for 15 min it was transferred dropwise into M1 at 0 °C. The resulting reaction mixture was stirred at 0 °C for 5 min and then stirred at room temperature for 20 h.

The solvent was removed under reduced pressure and the crude product was purified by column chromatography on SiO_2 (Cyhex/EtOAc; 20:1 to 5:1). The desired product **ItaCORM 1b** was obtained in form of a clear yellow oil in a yield of 131 mg (348 *µ*mol, 91%).

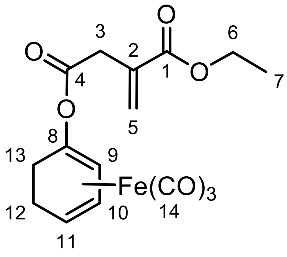

M ($C_{16}H_{16}FeO_7$) 376.14 g/mol.

Yield 131 mg (348 *µ*mol, 91%)

R_f 0.65 (SiO_2, Cyhex/EtOAc; 5:1).

^{1}H NMR (600 MHz, $CDCl_3$): δ [ppm] = 6.34 (d, *J* = 1.0 Hz, 1H, H-5a), 5.71 (d, *J* = 1.1 Hz, 1H, H-5b), 5.39 (dd, *J* = 4.5, 1.3 Hz, 1H, H-9), 5.10 (ddd, *J* = 5.7, 4.5, 1.0 Hz, 1H, H-10), 4.21 (q, *J* = 7.1 Hz, 2H, H-6), 3.37 – 3.29

(m, 2H, H-3), 3.11 – 3.07 (m, 1H, H-11), 2.18 – 2.11 (m, 1H, H-13a), 1.91 – 1.77 (m, 2H, H-12a, H-13b), 1.70 – 1.63 (m, 1H, H-12b), 1.29 (t, J = 7.1 Hz, 3H, H-7).

^{13}C NMR (150 MHz, $CDCl_3$): δ [ppm] = 211.3 (C-14), 210.7 (C-14), 169.1 (C-4), 166.1 (C-1), 133.7 (C-2), 128.8 (C-5), 103.6 (C-8), 80.7 (C-9 or C-10), 80.4 (C-9 or C-10), 61.2 (C-6), 60.5 (C-11), 38.1 (C-3), 26.8 (C-13), 24.2 (C-12), 14.3 (C-7).

HR-MS (ESI) calc.: $[M+Na]^+$ = 399.01377 amu found: 399.01365 amu.

FT-IR (ATR): ṽ [cm^{-1}] = 2986 (w), 2938 (w), 2914 (w), 2896 (w), 2859 (w), 2047 (vs), 1966 (vs), 1752 (m), 1718 (m), 1640 (w), 1473 (w), 1457 (w), 1443 (w), 1427 (w), 1380 (w), 1371 (w), 1330 (w), 1317 (m), 1242 (w), 1182 (m), 1165 (m), 1133 (m), 1111 (m), 1068 (w), 1029 (w), 1007 (w), 956 (w), 897 (w), 861 (w), 818 (w), 788 (w), 769 (w), 748 (w), 639 (w), 611 (m), 562 (m), 517 (w), 490 (w), 457 (w), 411 (w).

1.3.3 Synthesis of ItaCORM 2a

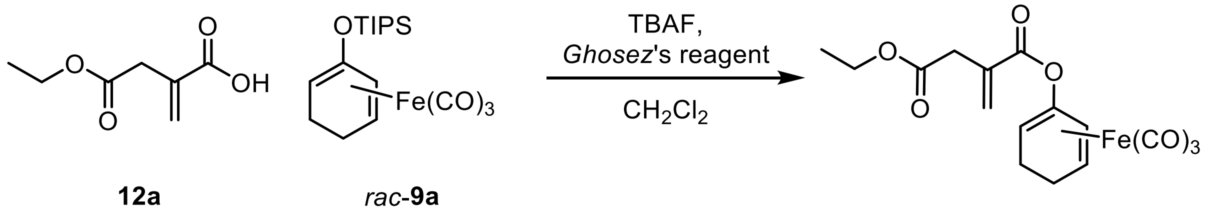

In a *Schlenk* tube, 4-ethyl itaconate (**12a**) (84.7 mg, 535 µmol, 1.40 eq.) was dissolved in CH_2Cl_2 (1.5 mL) and cooled to 0 °C. *Ghosez*'s reagent (79.0 µL, 573 µmol, 1.50 eq.) was added and the reaction mixture was stirred for 2.0 h (M1).

In a separate *Schlenk* tube, complex *rac*-**9a** (150 mg, 382 µmol, 1.00 eq.) was dissolved in CH_2Cl_2 (1.5 mL), cooled to 0 °C and TBAF (1 M in THF, 382 µL, 382 µmol, 1.00 eq.) was added dropwise (M2). Upon stirring the mixture for 15 min it was transferred dropwise into M1 at 0 °C. The resulting reaction mixture was stirred at 0 °C for 15 min and then stirred at room temperature for 20 h.

The solvent was removed under reduced pressure and the crude product was purified by column chromatography on SiO_2 (Cyhex/EtOAc; 6:1). The desired product **ItaCORM 2a** was obtained in form of a clear yellow oil in a yield of 140 mg (372 µmol, 97%).

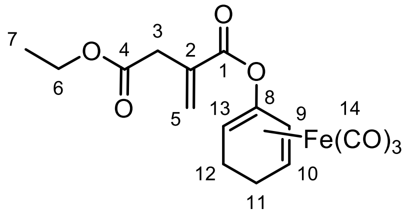

M ($C_{16}H_{16}FeO_7$) 376.14 g/mol.

Yield 140 mg (372 µmol, 97%)

R_f 0.73 (SiO_2, Cyhex/EtOAc; 5:1).

^{1}H NMR (500 MHz, $CDCl_3$): δ [ppm] = 6.42 (s, 1H, H-5a), 5.84 (s, 1H, H-5b), 5.55 (dd, J = 6.7, 2.0 Hz, 1H, H-9), 4.17 (qd, J = 7.2, 2.0 Hz, 2H, H-6), 3.44 – 3.32 (m, 3H, H-3, H-13), 2.87 (dt, J = 6.3, 2.9 Hz, 1H, H-10), 1.89 – 1.81 (m, 1H, H-12a), 1.81 – 1.72 (m, 1H, H-12b), 1.62 – 1.53 (m, 2H, H-11), 1.27 (t, J = 7.1 Hz, 3H, H-7).

^{13}C NMR (125 MHz, $CDCl_3$): δ [ppm] = 210.8 (C-14), 170.5 (C-4), 165.8 (C-1), 133.1 (C-2), 130.6 (C-5), 128.7 (C-8), 80.0 (C-9), 61.3 (C-6), 59.3 (C-13), 52.2 (C-10), 37.7 (C-3), 24.7 (C-12), 23.6 (C-11), 14.3 (C-7).

HR-MS (ESI) calc.: $[M+Na]^+$ = 399.01377 amu found: 399.01350 amu.

FT-IR (ATR): ṽ [cm^{-1}] = 2983 (w), 2935 (w), 2900 (w), 2855 (w), 2047 (vs), 1967 (vs), 1737 (m), 1641 (w), 1470 (w), 1459 (m), 1428 (w), 1393 (w), 1367 (w), 1337 (m), 1315 (m), 1255 (w), 1172 (m), 1124 (m), 1076 (w), 1032 (w), 961 (w), 949 (w), 930 (w), 879 (w), 855 (w), 807 (w), 775 (w), 736 (w), 685 (w), 620 (m), 572 (m), 515 (w), 504 (w), 461 (w).

1.3.4 Synthesis of ItaCORM 2b

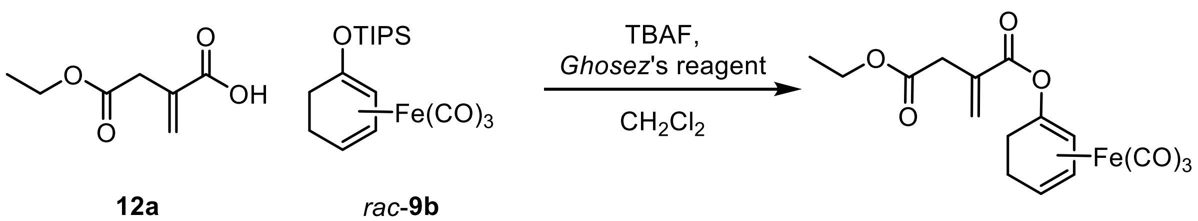

12a *rac*-**9b** **ItaCORM 2b**

In a *Schlenk* tube, 4-ethyl itaconate (**12a**) (84.7 mg, 535 µmol, 1.40 eq.) was dissolved in CH_2Cl_2 (1.3 mL) and cooled to 0 °C. *Ghosez*'s reagent (79.0 µL, 573 µmol, 1.50 eq.) was added and the reaction mixture was stirred for 1.5 h (M1).

In a separate *Schlenk* tube, complex *rac*-**9b** (150 mg, 382 µmol, 1.00 eq.) was dissolved in CH_2Cl_2 (1.3 mL), cooled to 0 °C and TBAF (1 M in THF, 382 µL, 382 µmol, 1.00 eq.) was

added dropwise (M2). Upon stirring the mixture for 15 min it was transferred dropwise into M1 at 0 °C. The resulting reaction mixture was stirred at 0 °C for 10 min and then stirred at room temperature for 20 h.

The solvent was removed under reduced pressure and the crude product was purified by column chromatography on SiO_2 (Cyhex/EtOAc; 6:1). The desired product **ItaCORM 2b** was obtained in form of a clear yellow oil in a yield of 134 mg (356 *µ*mol, 93%).

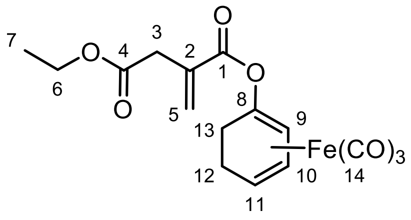

M ($C_{16}H_{16}FeO_7$) 376.14 g/mol.

Yield 134 mg (356 *µ*mol, 93%)

R_f 0.65 (SiO_2, Cyhex/EtOAc; 5:1).

^{1}H NMR (500 MHz, $CDCl_3$): δ [ppm] = 6.34 (d, *J* = 0.8 Hz, 1H, H-5a), 5.72 (d, *J* = 1.1 Hz, 1H, H-5b), 5.43 (dd, *J* = 4.5, 1.2 Hz, 1H, H-9), 5.13 (ddd, *J* = 6.1, 4.7, 0.9 Hz, 1H, H-10), 4.15 (q, *J* = 7.1 Hz, 2H, H-6), 3.33 (s, 2H, H-3), 3.11 (dddd, *J* = 6.3, 3.8, 2.3, 1.5 Hz, 1H, H-11), 2.19 – 2.12 (m, 1H, H-13a), 1.93 – 1.83 (m, 2H, H-12a, H-13b), 1.72 – 1.60 (m, 1H, H-12b), 1.26 (t, *J* = 7.1 Hz, 3H, H-7).

^{13}C NMR (125 MHz, $CDCl_3$): δ [ppm] = 211.3 (C-14), 169.1 (C-4), 164.6 (C-1), 133.9 (C-2), 129.0 (C-5), 103.8 (C-8), 80.8 (C-9 or C-10), 80.5 (C-9 or C-10), 61.1 (C-6), 60.5 (C-11), 37.9 (C-3), 26.8 (C-13), 24.3 (C-12), 14.3 (C-7).

HR-MS (ESI) calc.: $[M+Na]^+$ = 399.01377 amu found: 399.01368 amu.

FT-IR (ATR): ṽ [cm^{-1}] = 3671 (w), 2986 (w), 2938 (w), 2909 (w), 2861 (w), 2048 (vs), 1969 (vs), 1734 (m), 1642 (w), 1473 (w), 1427 (w), 1385 (w), 1370 (w), 1329 (w), 1317 (w), 1256 (w), 1183 (m), 1137 (m), 1069 (w), 1033 (w), 1006 (w), 952 (w), 867 (w), 812 (w), 758 (w), 612 (m), 561 (m), 518 (w), 485 (w), 461 (w).

1.3.5 Synthesis of ItaCORM 3a

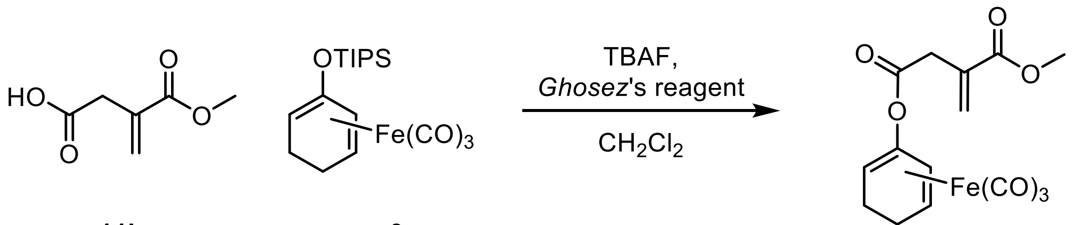

11b *rac*-**9a** **ItaCORM 3a**

In a *Schlenk* tube, 1-methyl itaconate (**11b**) (107 mg, 742 μmol, 1.43 eq.) was dissolved in CH_2Cl_2 (1.0 mL) and cooled to 0 °C. *Ghosez*'s reagent (120 μL, 906 μmol, 1.74 eq.) was added and the reaction mixture was stirred for 1 h (M1).
In a separate *Schlenk* tube, complex *rac*-**9a** (204 mg, 520 μmol, 1.00 eq.) was dissolved in CH_2Cl_2 (1.0 mL), cooled to 0 °C and TBAF (1 M in THF, 520 μL, 520 μmol, 1.0 eq.) was added dropwise (M2). Upon stirring the mixture for 10 min it was transferred dropwise into M1 at 0 °C. The resulting reaction mixture was stirred at 0 °C for 10 min and then stirred at room temperature for 21 h.
The solvent was removed under reduced pressure and the crude product was purified by column chromatography on SiO_2 (Cyhex/EtOAc; 20:1 to 7:1). The desired product **ItaCORM 3a** was obtained in form of a clear yellow oil in a yield of 63 mg (170 μmol, 33%).

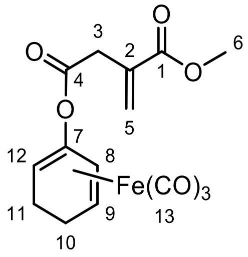

M ($C_{15}H_{14}FeO_7$) 362.12 g/mol.

Yield 63 mg (170 μmol, 33%)

R_f 0.57 (SiO_2, Cyhex/EtOAc; 6:1).

^{1}H NMR (500 MHz, $CDCl_3$): δ [ppm] = 6.38 (s, 1H, H-5a), 5.79 (s, 1H, H-5b), 5.52 (dd, J = 6.6, 1.5 Hz, 1H, H-8), 3.78 (s, 3, H-6), 3.49 – 3.41 (m, 2H, H-3), 3.38 (dt, J = 3.8, 2.3 Hz, 1H, H-12), 2.87 – 2.83 (m, 1H, H-9), 1.86 – 1.79 (m, 1H, H-11a), 1.79 – 1.71 (m, 1H, H-11b), 1.58 – 1.48 (m, 2H, H-10).

^{13}C NMR (125 MHz, $CDCl_3$): δ [ppm] = 210.8 (C-13), 170.2 (C-4), 166.5 (C-1), 133.1 (C-2), 129.4 (C-5), 128.5 (C-7), 79.8 (C-8), 59.1 (C-12), 52.4 (C-9), 52.2 (C-6), 37.9 (C-3), 24.7 (C-11), 23.6 (C-10).

HR-MS (ESI) calc.: $[M+Na]^+$ = 384.99812 amu found: 384.99806 amu.

FT-IR (ATR): $\tilde{\nu}$ [cm^{-1}] = 3010 (w), 2954 (w), 2940 (w), 2902 (w), 2881 (w), 2857 (w), 2046 (s), 1962 (vs), 1763 (w), 1744 (m), 1726 (m), 1646 (w), 1474 (w), 1458 (w), 1438 (w), 1395 (w), 1356 (w), 1321 (w), 1263 (m), 1216

(w), 1164 (m), 1110 (m), 1076 (w), 1010 (w), 996 (w), 974 (w), 924 (w), 899 (w), 864 (w), 840 (w), 818 (w), 770 (w), 679 (w), 619 (m), 608 (m), 572 (m), 513 (w), 461 (w).

1.3.6 Synthesis of ItaCORM 3b

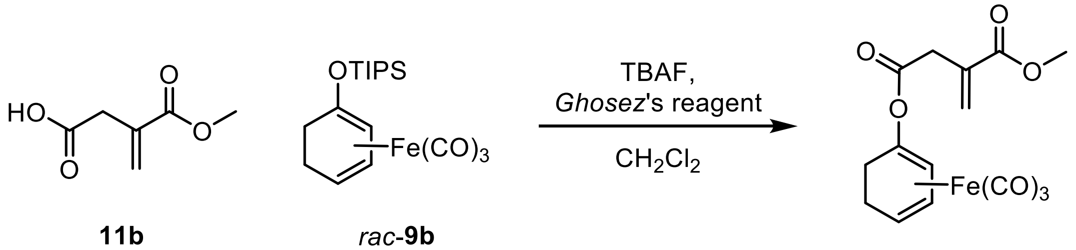

11b *rac*-**9b** **ItaCORM 3b**

In a *Schlenk* tube, 1-methyl itaconate (**11b**) (56 mg, 389 μmol, 1.47 eq.) was dissolved in CH_2Cl_2 (1.0 mL) and cooled to 0 °C. *Ghosez*'s reagent (51 μL, 389 μmol, 1.47 eq.) was added and the reaction mixture was stirred for 1 h (M1).

In separate *Schlenk* tube, complex *rac*-**9b** (104 mg, 265 μmol, 1.00 eq.) was dissolved in CH_2Cl_2 (1.3 mL), cooled to 0 °C and TBAF (1 M in THF, 280 μL, 280 μmol, 1.06 eq.) was added dropwise (M2). Upon stirring the mixture for 15 min it was transferred dropwise into M1 at 0 °C. The resulting reaction mixture was stirred at 0 °C for 10 min and then stirred at room temperature for 22 h.

The solvent was removed under reduced pressure and the crude product was purified by column chromatography on SiO_2 (Cyhex/EtOAc; 6:1). The desired product **ItaCORM 3b** was obtained in form of a clear yellow oil in a yield of 76 mg (210 μmol, 79%). The product crystallized upon storage.

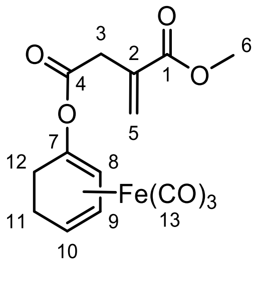

M ($C_{15}H_{14}FeO_7$) 362.12 g/mol.

Yield 76 mg (210 μmol, 79%)

R_f 0.42 (SiO_2, Cyhex/EtOAc; 5:1).

^{1}H NMR (600 MHz, $CDCl_3$): δ [ppm] = 6.35 (s, 1H, H-5a), 5.82 – 5.57 (m, 1H, H-5b), 5.39 (d, *J* = 4.4 Hz, 1H, H-8), 5.11 (t, *J* = 5.5 Hz, 1H, H-9), 3.75 (s, 3H, H-6), 3.39 – 3.26 (m, 2H, H-3), 3.12 – 3.06 (m, 1H, H-10), 2.19 – 2.09 (m, 1H, H-12a), 1.92 – 1.75 (m, 2H, H-11a, H-12b), 1.71 – 1.62 (m, 1H, H-11b).

^{13}C NMR (150 MHz, $CDCl_3$): δ [ppm] = 211.3 (C-13), 169.0 (C-4), 166.6 (C-1), 133.4 (C-2), 129.2 (C-5), 103.6 (C-7), 80.8 (C-8 or C-9), 80.3 (C-8 or C-9), 60.5 (C-10), 52.3 (C-6), 38.1 (C-3), 26.8 (C-12), 24.2 (C-11).

HR-MS (ESI) calc.: $[M+Na]^+$ = 384.99812 amu found: 384.99793 amu.

FT-IR (ATR): ṽ [cm^{-1}] = 3008 (w), 2953 (w), 2917 (w), 2894 (w), 2858 (w), 2044 (s), 1957 (s), 1749 (s), 1723 (s), 1640 (m), 1468 (m), 1455 (m), 1439 (m), 1384 (m), 1339 (m), 1318 (m), 1262 (m), 1244 (m), 1202 (m), 1170 (s), 1132 (s), 1109 (s), 1067 (m), 1030 (w), 1006 (m), 956 (m), 913 (w), 865 (m), 840 (m), 817 (m), 791 (w), 768 (w), 750 (w), 711 (w), 638 (m), 609 (s), 560 (s), 518 (m).

m.p. 33.1 – 35.4 °C.

1.3.7 Synthesis of ItaCORM 4a

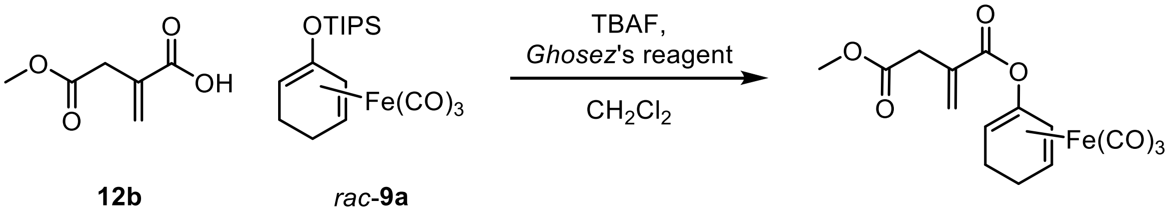

12b *rac*-**9a** **ItaCORM 4a**

In a *Schlenk* tube, 4-methyl itaconate (**12b**) (~90%, 64 mg, 400 *µ*mol, 1.52 eq.) was dissolved in CH_2Cl_2 (1.0 mL) and cooled to 0 °C. *Ghosez*'s reagent (51 *µ*L, 389 *µ*mol, 1.48 eq.) was added and the reaction mixture was stirred for 2.0 h (M1).

In a separate *Schlenk* tube, complex *rac*-**9a** (103 mg, 263 *µ*mol, 1.00 eq.) was dissolved in CH_2Cl_2 (1.0 mL), cooled to 0 °C and TBAF (1 M in THF, 0.29 mL, 0.29 mmol, 1.1 eq.) was added dropwise (M2). Upon stirring the mixture for 10 min it was transferred dropwise into M1 at 0 °C. The resulting reaction mixture was stirred at 0 °C for 30 min and then stirred at room temperature for 16.5 h.

The solvent was removed under reduced pressure and the crude product was purified by column chromatography on SiO_2 (Cyhex/EtOAc; 6:1). The desired product **ItaCORM 4a** was obtained in form of a clear yellow oil in a yield of 77 mg (213 *µ*mol, 81%).

M ($C_{15}H_{14}FeO_7$) 362.12 g/mol.

Yield 77 mg (213 *µ*mol, 81%)

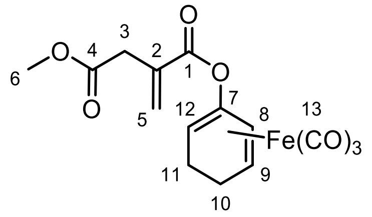

**R_f**	0.41 (SiO_2, Cyhex/EtOAc; 5:1).
**^{1}H NMR**	(500 MHz, $CDCl_3$): δ [ppm] = 6.43 (s, 1H, H-5a), 5.85 (s, 1H, H-5b), 5.56 (dd, J = 6.6, 1.8 Hz, 1H, H-8), 3.71 (s, 3H, H-6), 3.57 – 3.26 (m, 3H, H-3, H-12), 2.88 (dt, J = 6.3, 3.0 Hz, 1H, H-9), 1.95 – 1.72 (m, 1H, H-12), 1.71 - 1.44 (m, 2H, H-11).
**^{13}C NMR**	(125 MHz, $CDCl_3$): δ [ppm] = 210.8 (C-13), 170.9 (C-4), 165.7 (C-1), 132.9 (C-2), 130.8 (C-5), 128.7 (C-7), 80.0 (C-8), 59.3 (C-12), 52.3 (C-9), 52.3 (C-6), 37.4 (C-3), 24.7 (C-11), 23.6 (C-10).
**HR-MS (ESI)**	calc.: $[M+Na]^+$ = 384.99812 amu found: 384.99808 amu.
**FT-IR**	(ATR): ṽ [cm^{-1}] = 3007 (w), 2954 (w), 2933 (w), 2906 (w), 2856 (w), 2047 (s), 1966 (vs), 1741 (m), 1641 (w), 1459 (w), 1437 (w), 1401 (w), 1343 (w), 1317 (w), 1256 (w), 1171 (m), 1124 (m), 1074 (w), 1017 (w), 997 (w), 958 (w), 945 (w), 928 (w), 896 (w), 872 (w), 843 (w), 808 (w), 777 (w), 745 (w), 686 (w), 619 (m), 610 (m), 572 (m), 515 (w), 461 (w).

1.3.8 Synthesis of ItaCORM 4b

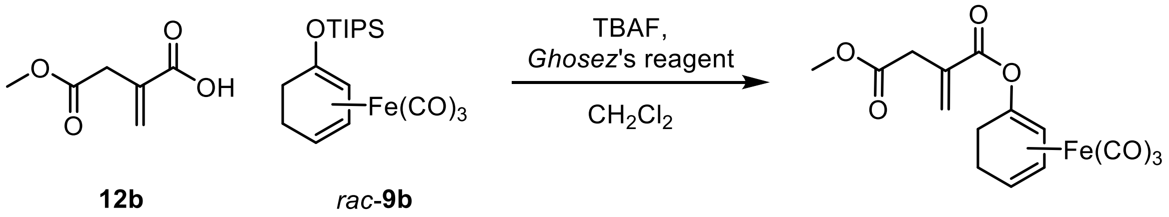

In a *Schlenk* tube, 1-methyl itaconate (**12b**) (~90%, 62 mg, 387 µmol, 1.51 eq.) was dissolved in CH_2Cl_2 (1.0 mL) and cooled to 0 °C. *Ghosez*'s reagent (51 µL, 389 µmol, 1.51 eq.) was added and the reaction mixture was stirred for 1 h (M1).

In a separate *Schlenk* tube, complex *rac*-**9b** (101 mg, 257 µmol, 1.00 eq.) was dissolved in CH_2Cl_2 (1.0 mL), cooled to 0 °C and TBAF (1 M in THF, 0.28 mL, 280 µmol, 1.09 eq.) was added dropwise (M2). Upon stirring the mixture for 10 min it was transferred dropwise into M1 at 0 °C. The resulting reaction mixture was stirred at 0 °C for 1 h and then stirred at room temperature for 23 h.

The solvent was removed under reduced pressure and the crude product was purified by column chromatography on SiO_2 (Cyhex/EtOAc; 6:1). The desired product **ItaCORM 4b** was

obtained in form of a clear yellow oil in a yield of 69 mg (191 µmol, 74%). The product crystallized upon storage.

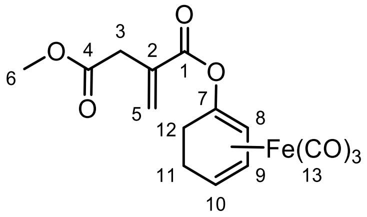

M ($C_{15}H_{14}FeO_7$) 362.12 g/mol.

Yield 69 mg (191 µmol, 74%)

R*f* 0.40 (SiO_2, Cyhex/EtOAc; 5:1).

^{1}H NMR (500 MHz, $CDCl_3$): δ [ppm] = 6.34 (s, 1H, H-5a), 5.72 (d, *J* = 1.1 Hz, 1H, H-5b), 5.43 (dd, *J* = 4.5, 1.2 Hz, 1H, H-8), 5.23 – 4.99 (m, 1H, H-9), 3.69 (s, 3H, H-6), 3.35 (d, *J* = 1.1 Hz, 2H, H-3), 3.11 (dddd, *J* = 6.3, 3.8, 2.4, 1.5 Hz, 1H, H-10), 2.23 – 2.10 (m, 1H, H-12a), 1.97 – 1.81 (m, 2H, H-11a, H-12b), 1.75 – 1.66 (m, 1H, H-11b).

^{13}C NMR (125 MHz, $CDCl_3$): δ [ppm] 211.2 (C13), 171.1 (C-4), 164.6 (C-1), 133.8 (C-2), 129.2 (C-5), 103.8 (C-7), 80.8 (C-8), 80.5 (C-9), 60.5 (C-10), 52.2 (C-1), 37.6 (C-3), 26.8 (C-12), 24.3 (C-11).

HR-MS (ESI) calc.: $[M+Na]^+$ = 384.99812 amu found: 384.99803 amu.

FT-IR (ATR): $\tilde{v}$ [cm^{-1}] = 3007 (w), 2953 (w), 2919 (w), 2869 (w), 2858 (w), 2045 (s), 1957 (s), 1729 (s), 1641 (w), 1471 (w), 1456 (w), 1436 (m), 1404 (w), 1383 (w), 1339 (m), 1319 (m), 1261 (m), 1179 (m), 1134 (s), 1067 (w), 1006 (m), 955 (m), 942 (m), 905 (m), 865 (m), 839 (w), 811 (m), 760 (w), 740 (w), 637 (m), 611 (s), 560 (s), 540 (w), 517 (w).

1.4 In situ quantification of CO-release

1.4.1 Headspace-GC-system

For the CO-release quantification a *Thermo Scientific* Trace 1300 headspace gas chromatograph equipped with a TriPlus RSH autosampler was used. The detector was a thermal conductivity detector (TCD). The system was equipped with a Shin carbon ST 10/120 1.0 mm x 2 m 1/16'' OD silico column. For the programming of the system *Chromeleon*® 7 Data System Software was used.

1.4.2 Gas-chromatography conditions for GC-Headspace system

Helium was used as carrier gas with a flow of 15 mL/min. Injector temperature: 200 °C. Split flow 150 mL/min, split rate 10. Detector temperature: 200 °C.

Method description: 0 – 2.5 min at 35 °C, then heated to 70 °C with a 20 °C/min rate, kept 1 min at 70 °C and then cooled to 35 °C with a 20 °C/min rate and kept 1 min at 35 °C. Injection volume: 50 μL

1.4.3 Calibration

Eight headspace vials (VWR, cat. No. 548-0090, vial headspace clear glass, 10 mL) were charged with DMSO (0.2 mL) and phosphate buffer solution (1.0 mL, pH = 7.4). The vials were sealed with gas-tight silicone/PTFE septa aluminium crimp caps (BGB Analytics, Cat. No. 20030500) and degassed with N_2.

A defined gas volume of the vial was substituted for CO (0.00 mL, 0.05 mL, 0.10 mL, 0.25 mL, 0.50 mL, 1.00 mL, 1.50 mL, 2.00 mL). The vials were then equilibrated at 37 °C for 10 min and the composition of the gas phase was subsequently determined by headspace GC analysis. The respective measurements were repeated three times and a calibration curve was then generated.

1.4.4 In situ quantification of the CO-release of ItaCORMs

The respective complex (36 μmol) was dissolved in DMSO (0.2 mL) and phosphate buffer solution (1.0 mL, pH = 7.4). Pig liver esterase (PLE) (15 mg, 24 units/mg) was added and the mixture was stirred at 37 °C. The amount of CO released was determined *via* headspace GC analysis using the previously generated calibration curve. Control samples without addition of PLE were analyzed according to the same procedure.

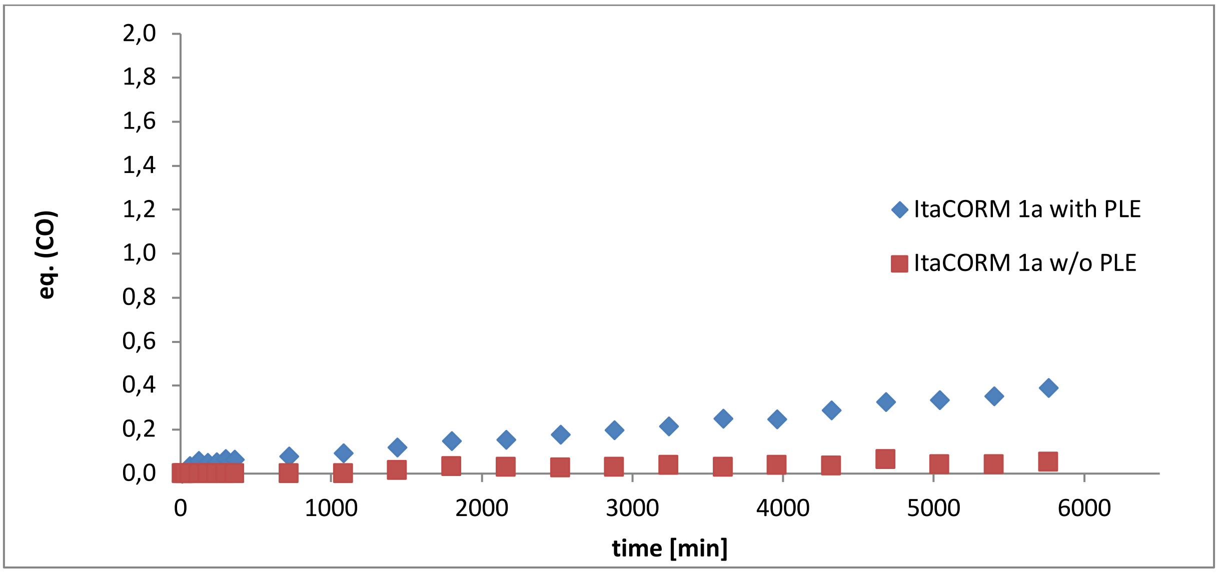

Fig. 1: Enzyme-triggered CO-release of **ItaCORM 1a** with PLE (blue) and without PLE (red).

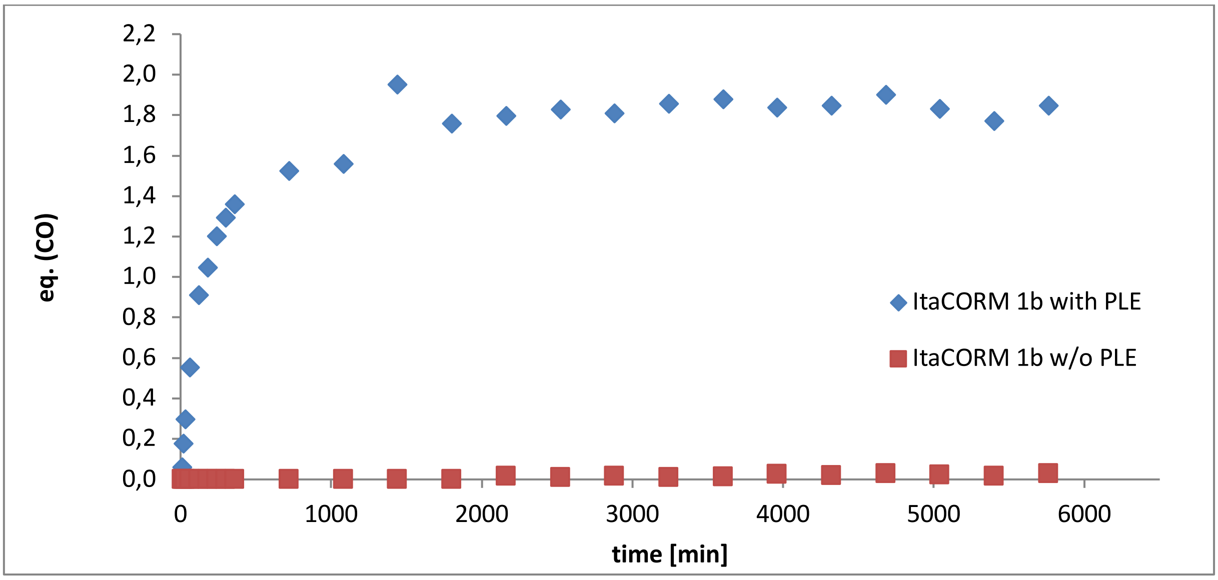

Fig. 2: Enzyme-triggered CO-release of **ItaCORM 1b** with PLE (blue) and without PLE (red).

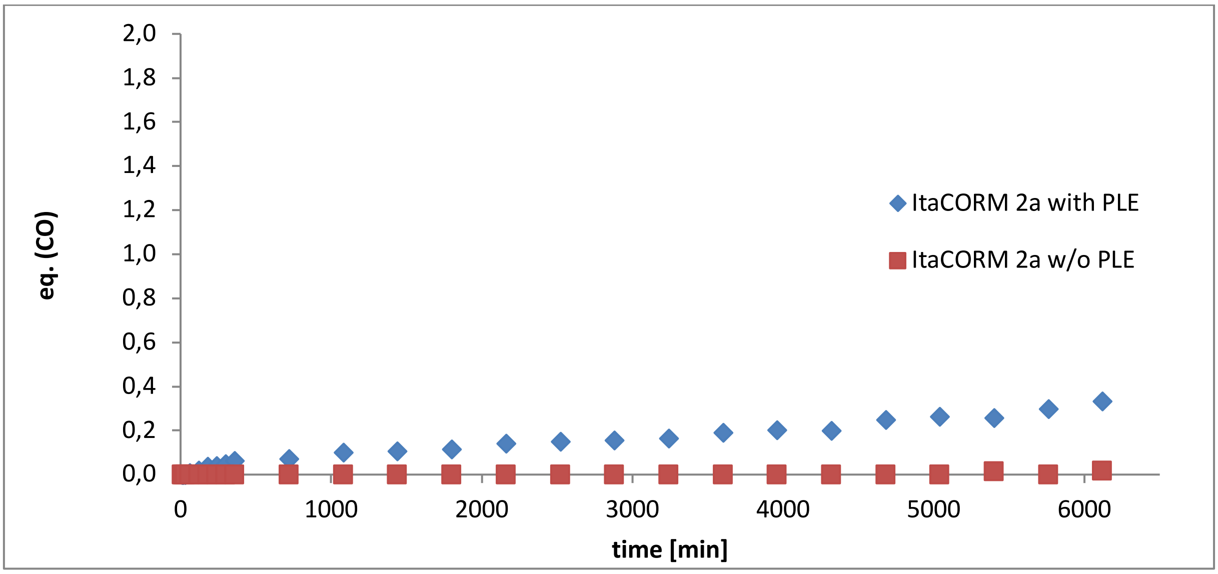

Fig. 3: Enzyme-triggered CO-release of **ItaCORM 2a** with PLE (blue) and without PLE (red).

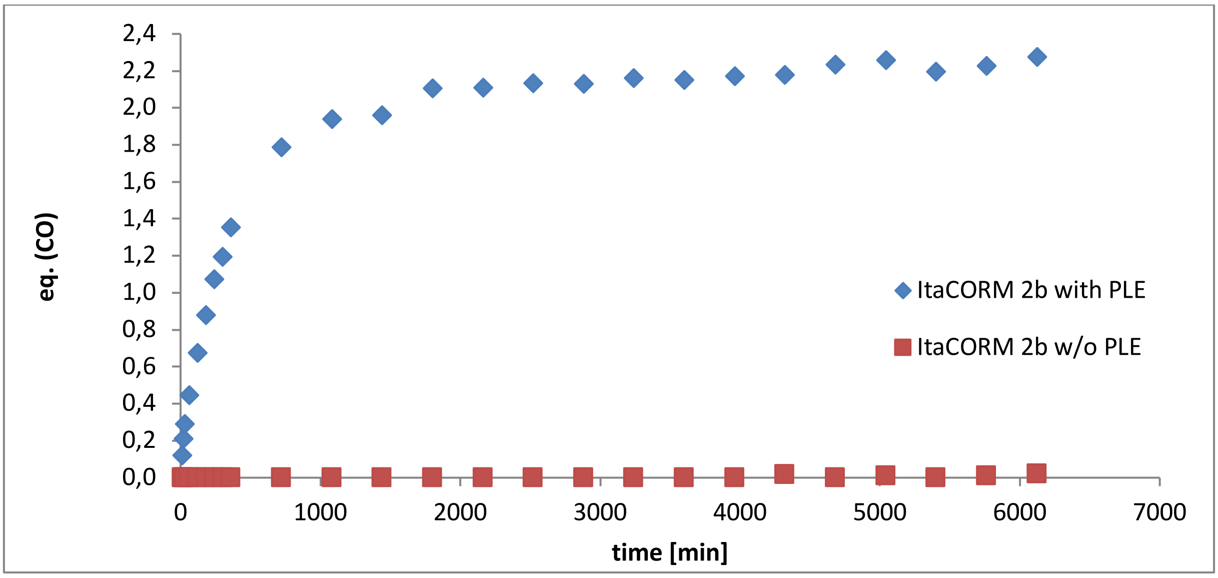

Fig. 4: Enzyme-triggered CO-release of **ItaCORM 2b** with PLE (blue) and without PLE (red).

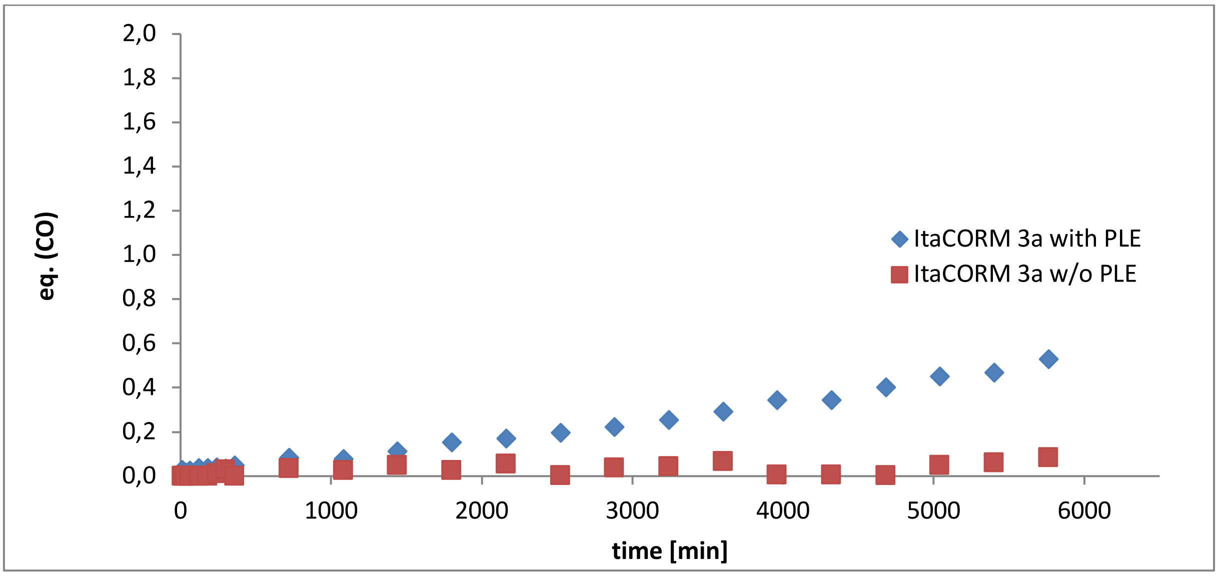

Fig. 5: Enzyme-triggered CO-release of **ItaCORM 3a** with PLE (blue) and without PLE (red).

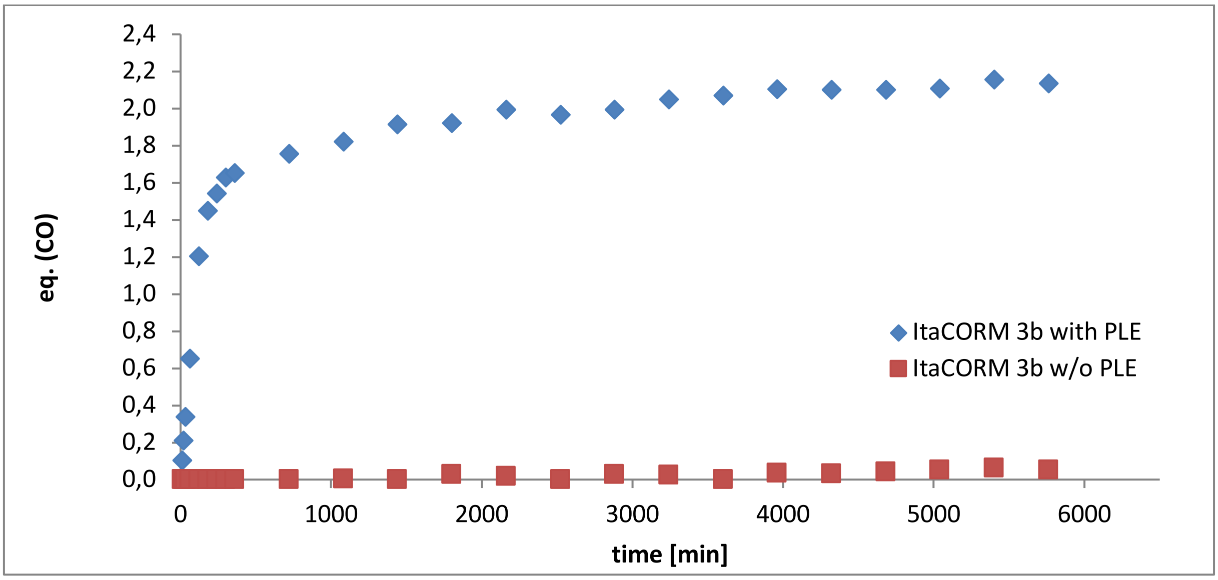

Fig. 6: Enzyme-triggered CO-release of **ItaCORM 3b** with PLE (blue) and without PLE (red).

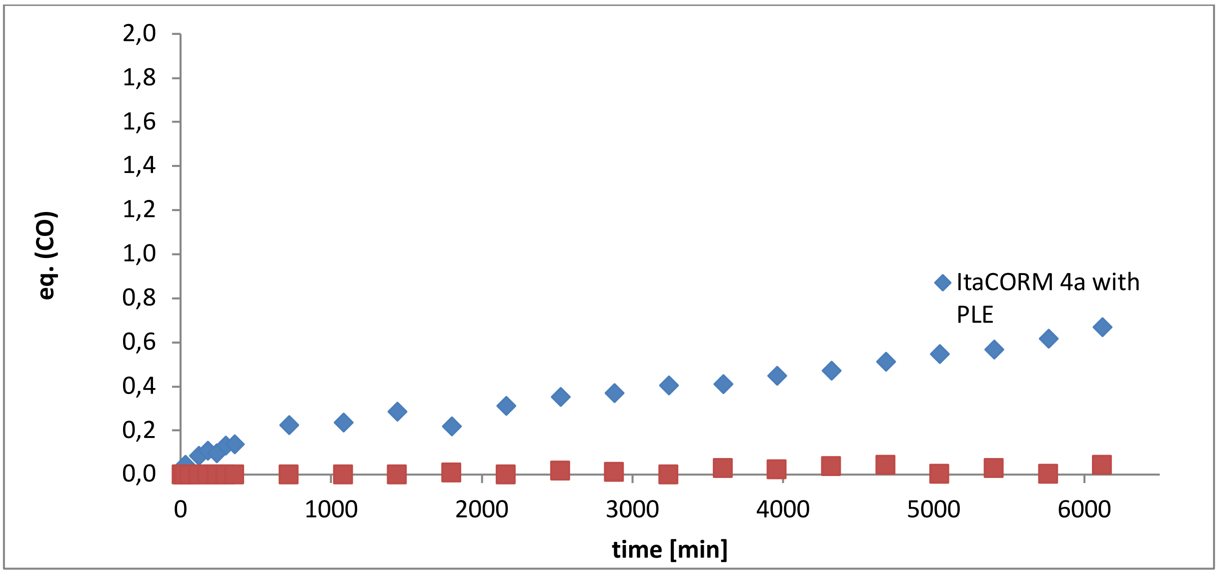

Fig. 7: Enzyme-triggered CO-release of **ItaCORM 4a** with PLE (blue) and without PLE (red).

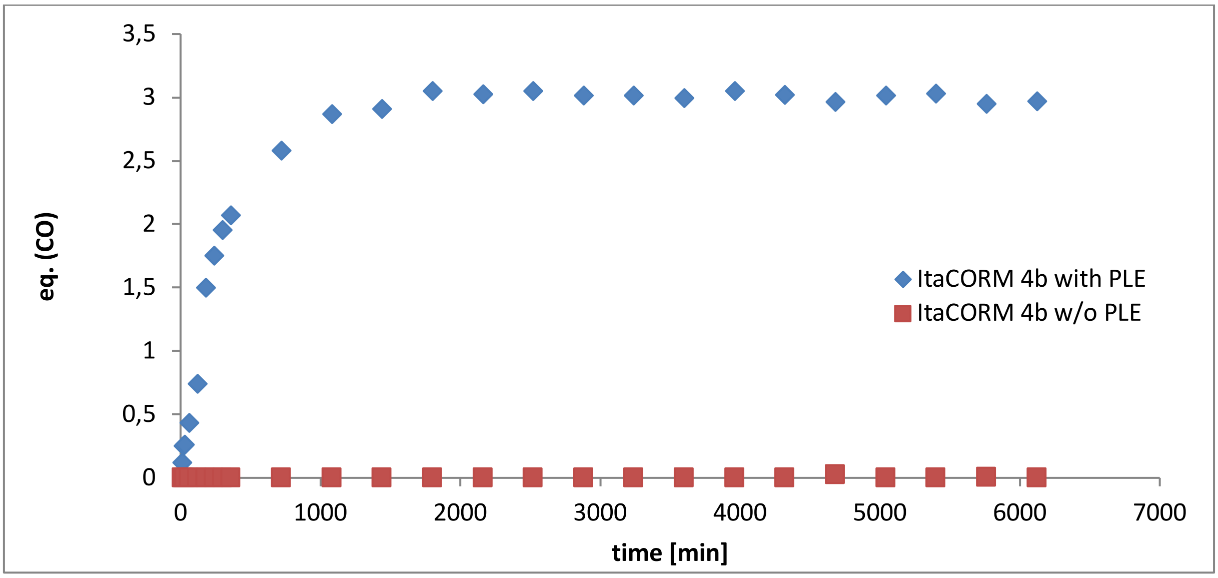

Fig. 8: Enzyme-triggered CO-release of **ItaCORM 4b** with PLE (blue) and without PLE (red).

1.5 NMR-Spectra

1.5.1 ^{1}H (300 MHz, $CDCl_3$) and ^{13}C (75 MHz, $CDCl_3$) spectra of diethyl itaconate (DEI, 13)

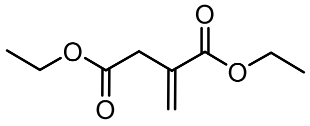

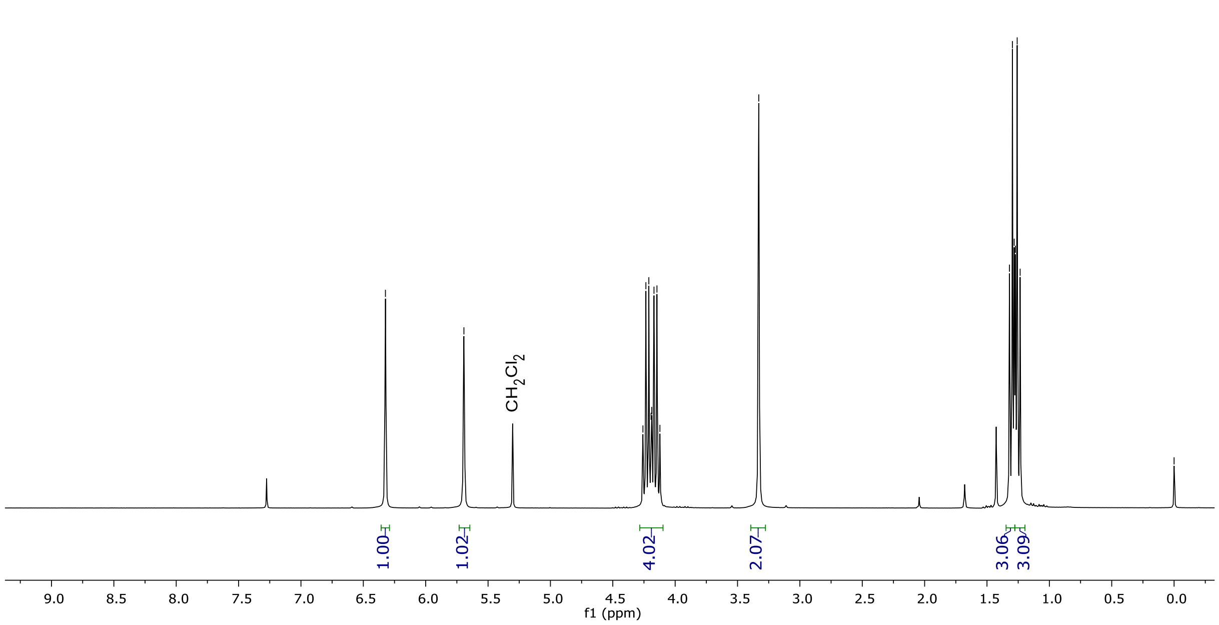

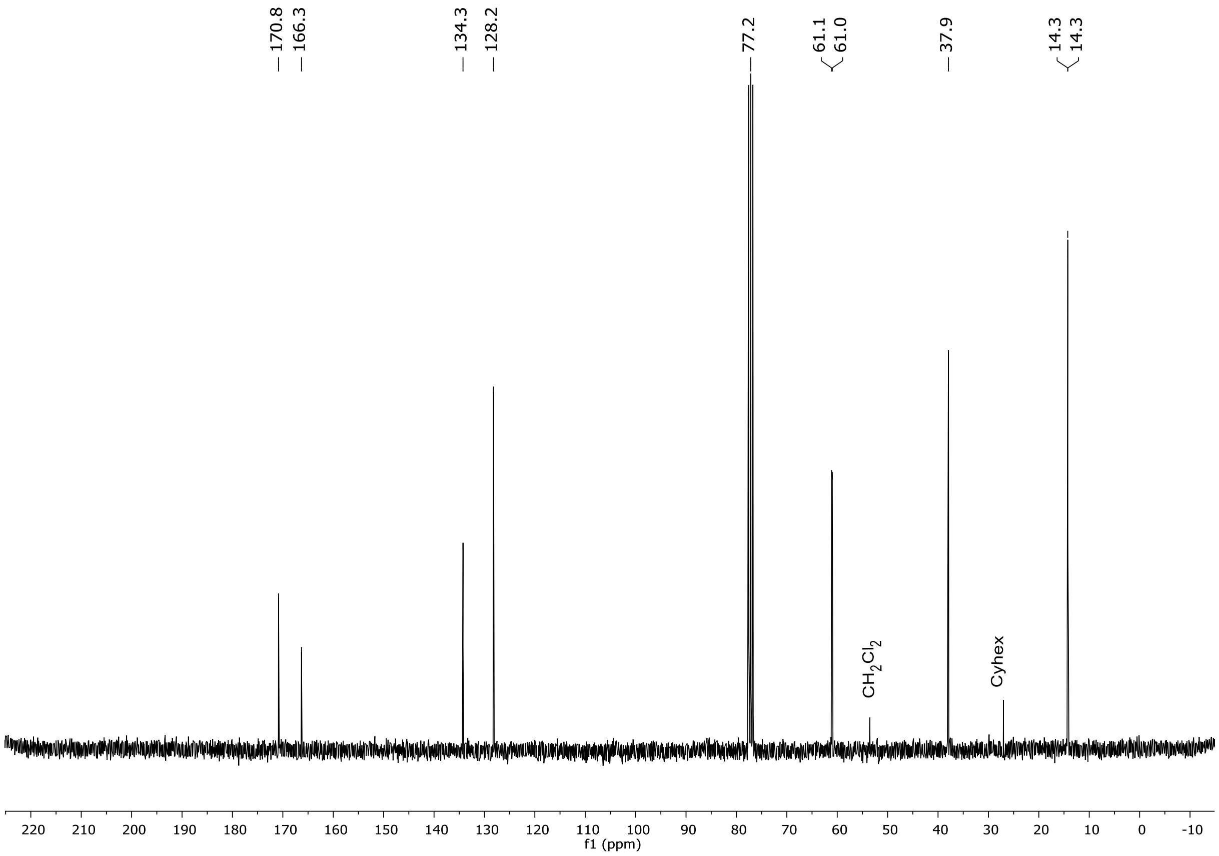

1.5.2 ^{1}H (300 MHz, $CDCl_3$) and ^{13}C (75 MHz, $CDCl_3$) spectra of 1-ethyl itaconate (11a)

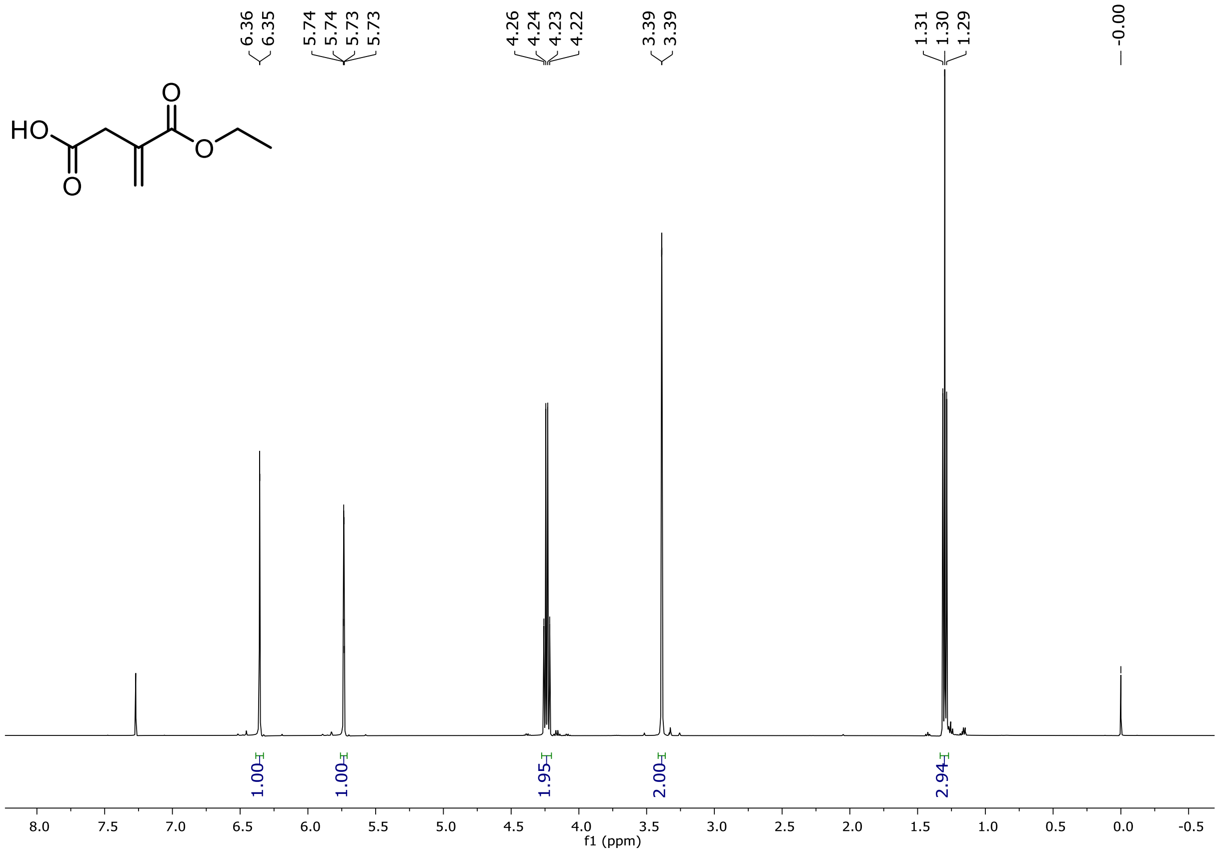

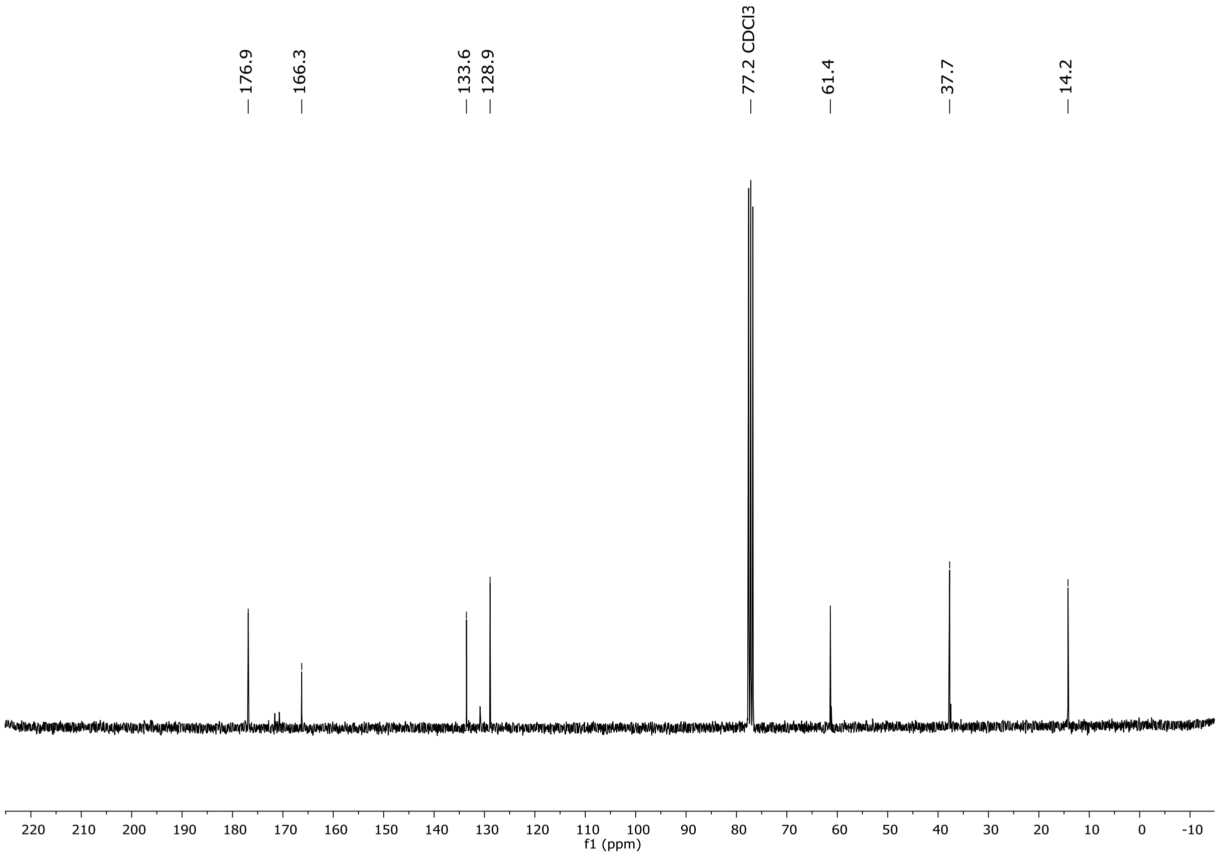

1.5.3 ^{1}H (500 MHz, $CDCl_3$) and ^{13}C (125 MHz, $CDCl_3$) spectra of ItaCORM 1a

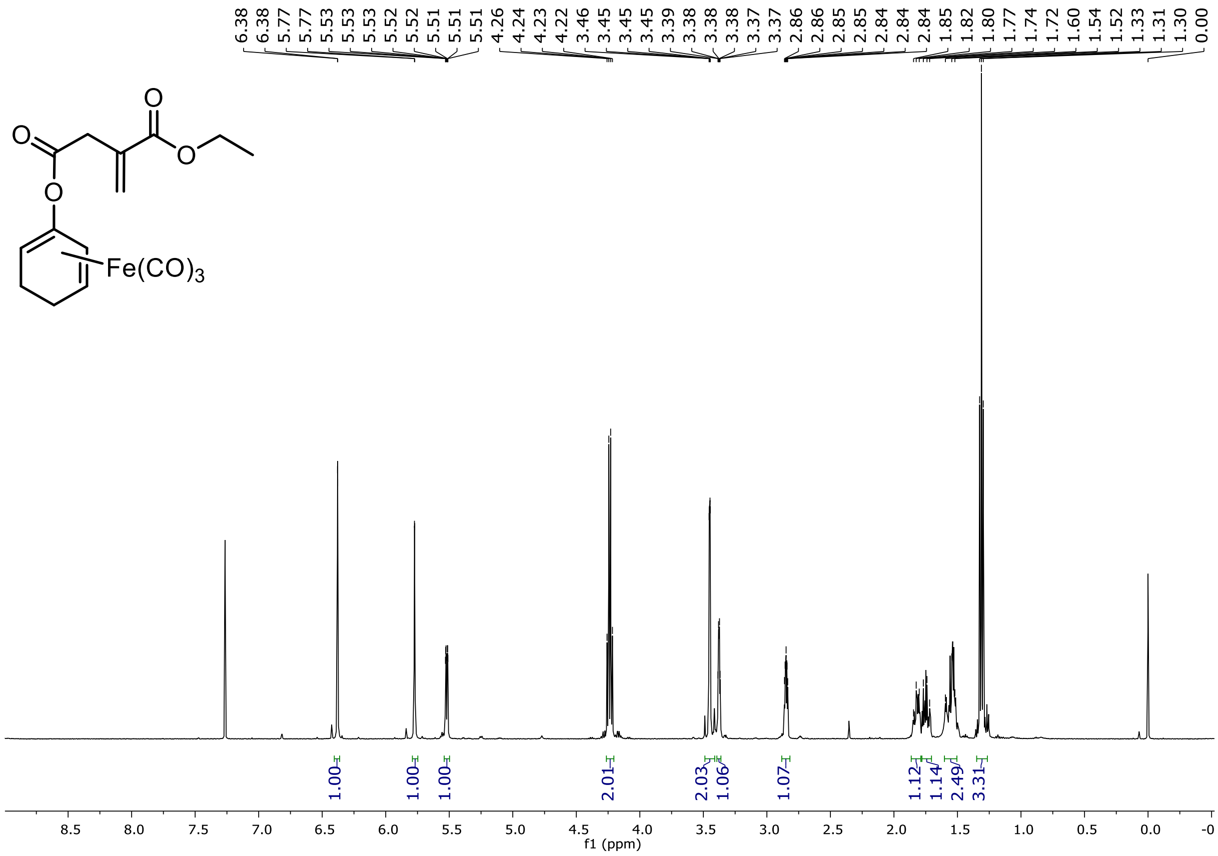

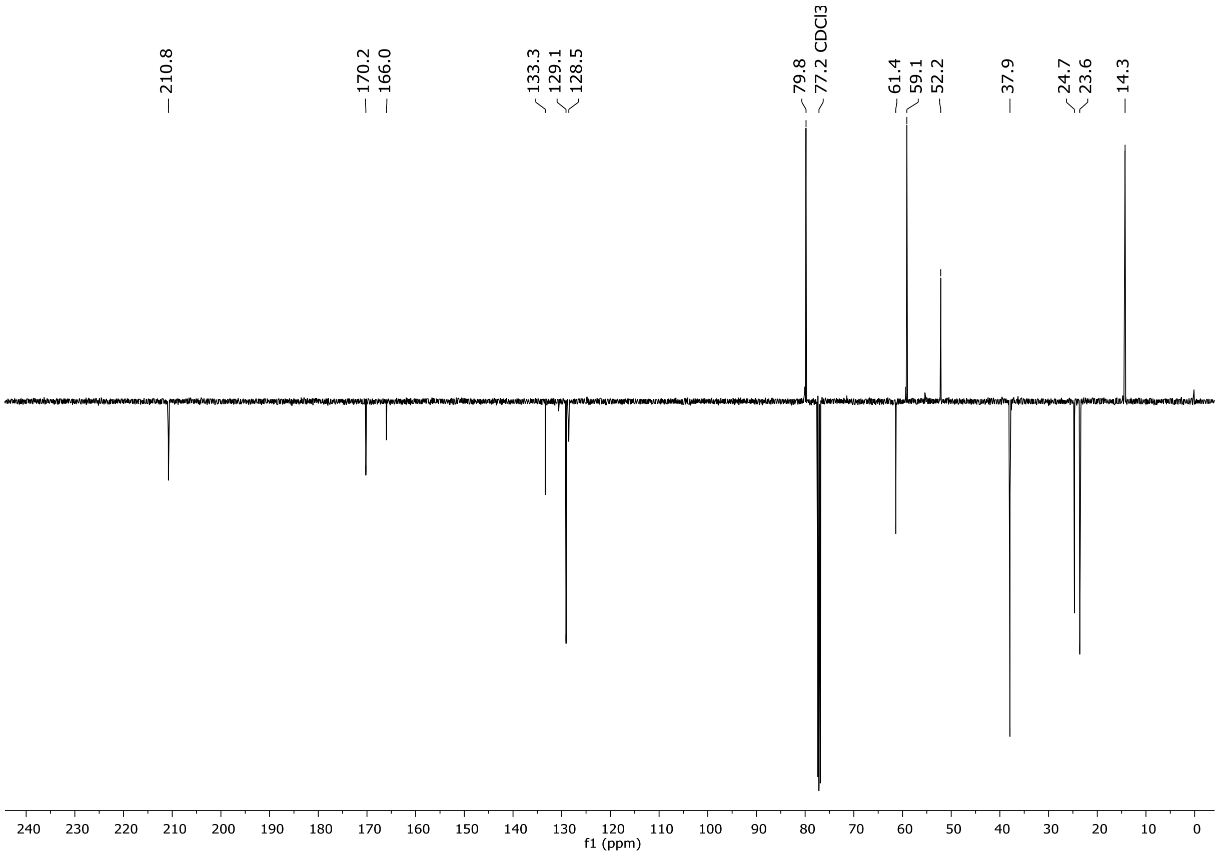

1.5.4 ^{1}H (600 MHz, $CDCl_3$) and ^{13}C (150 MHz, $CDCl_3$) spectra of ItaCORM 1b

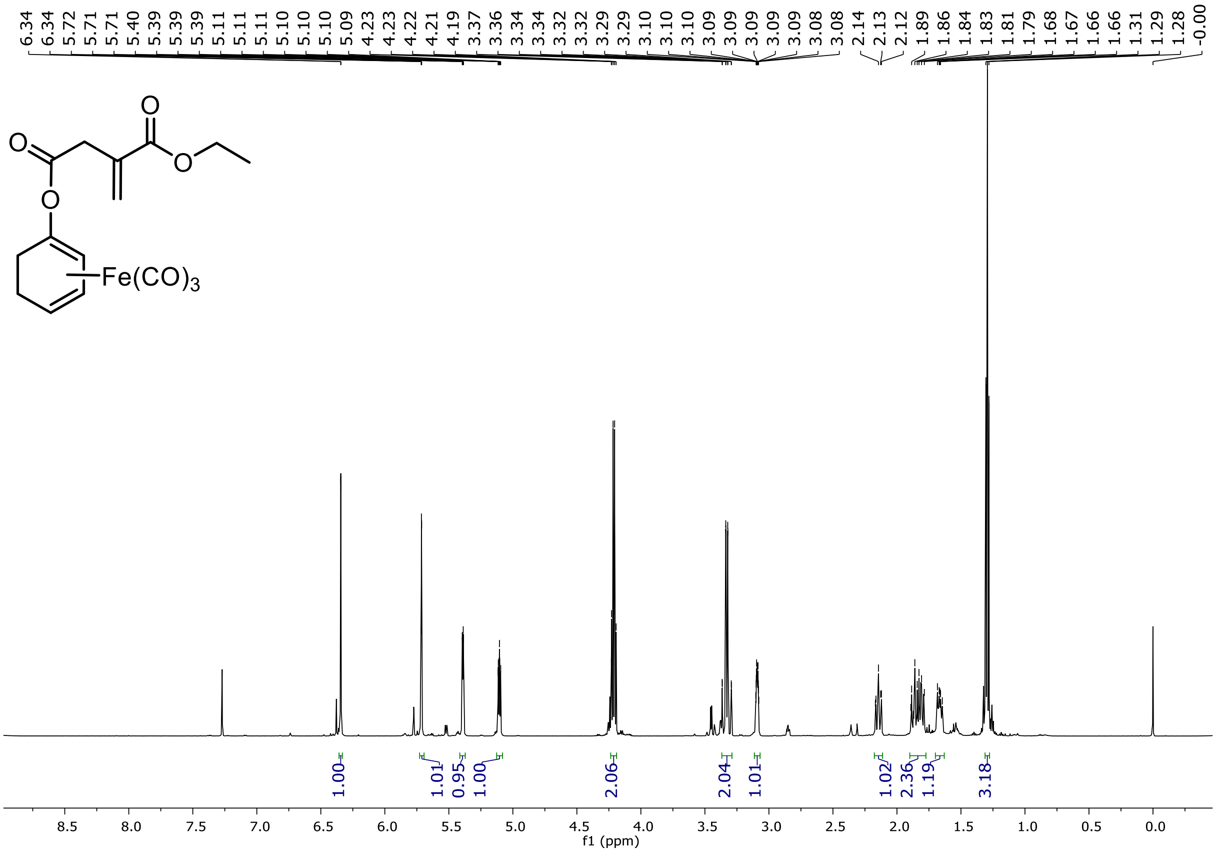

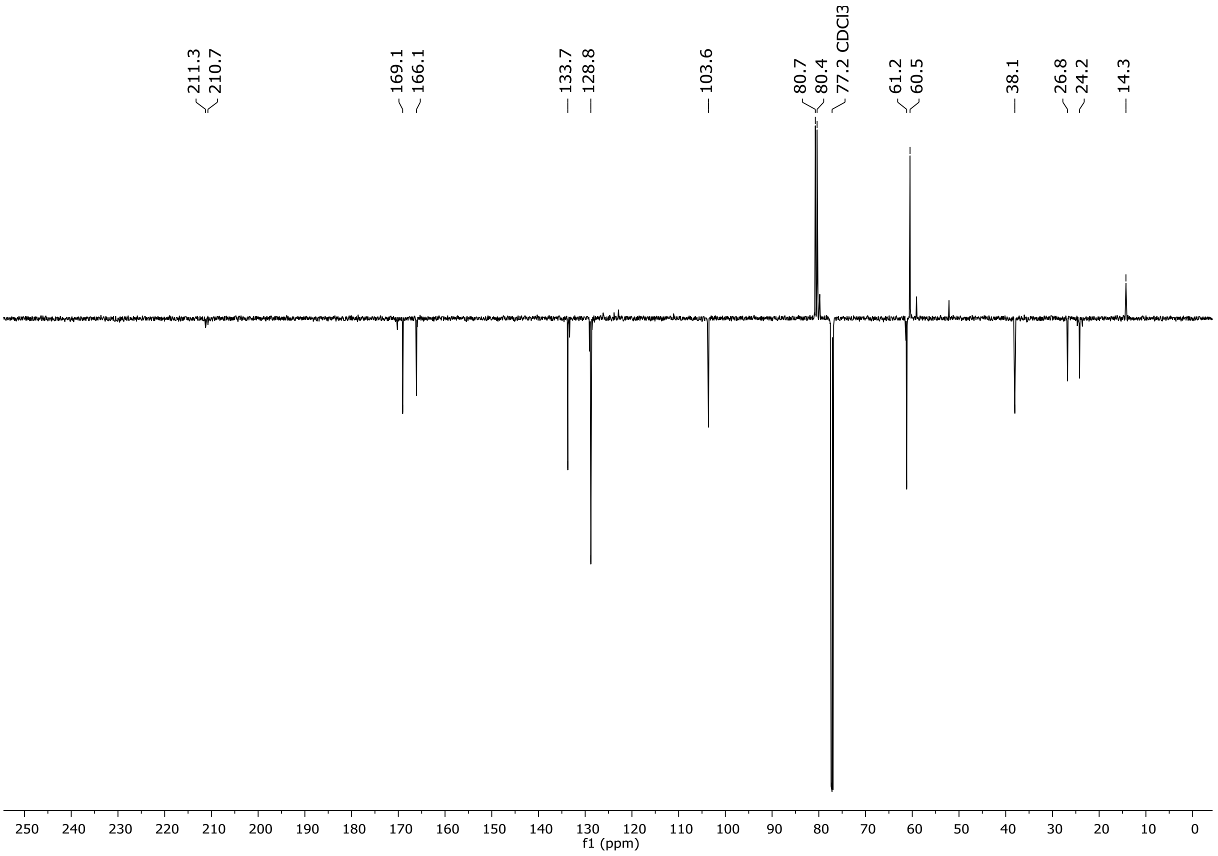

1.5.5 ^{1}H (500 MHz, $CDCl_3$) and ^{13}C (125 MHz, $CDCl_3$) spectra of ItaCORM 2a

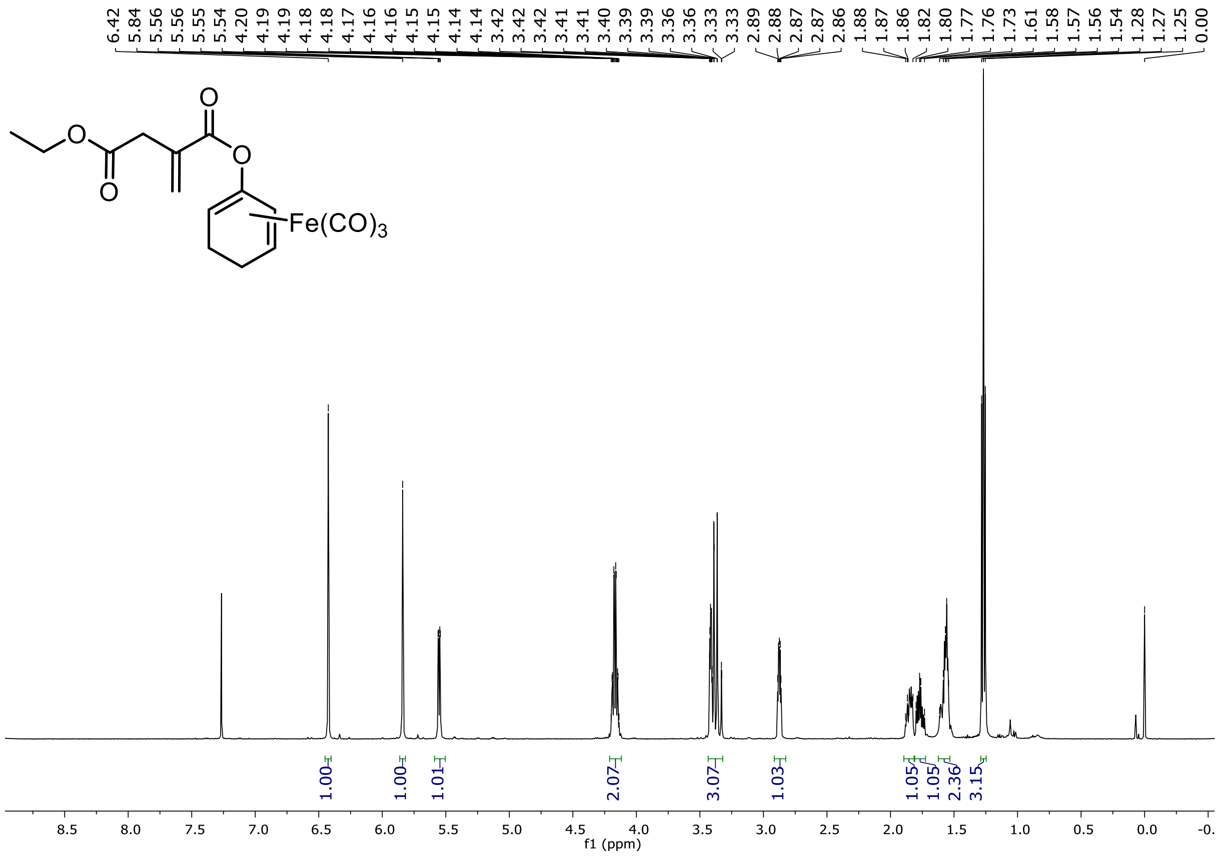

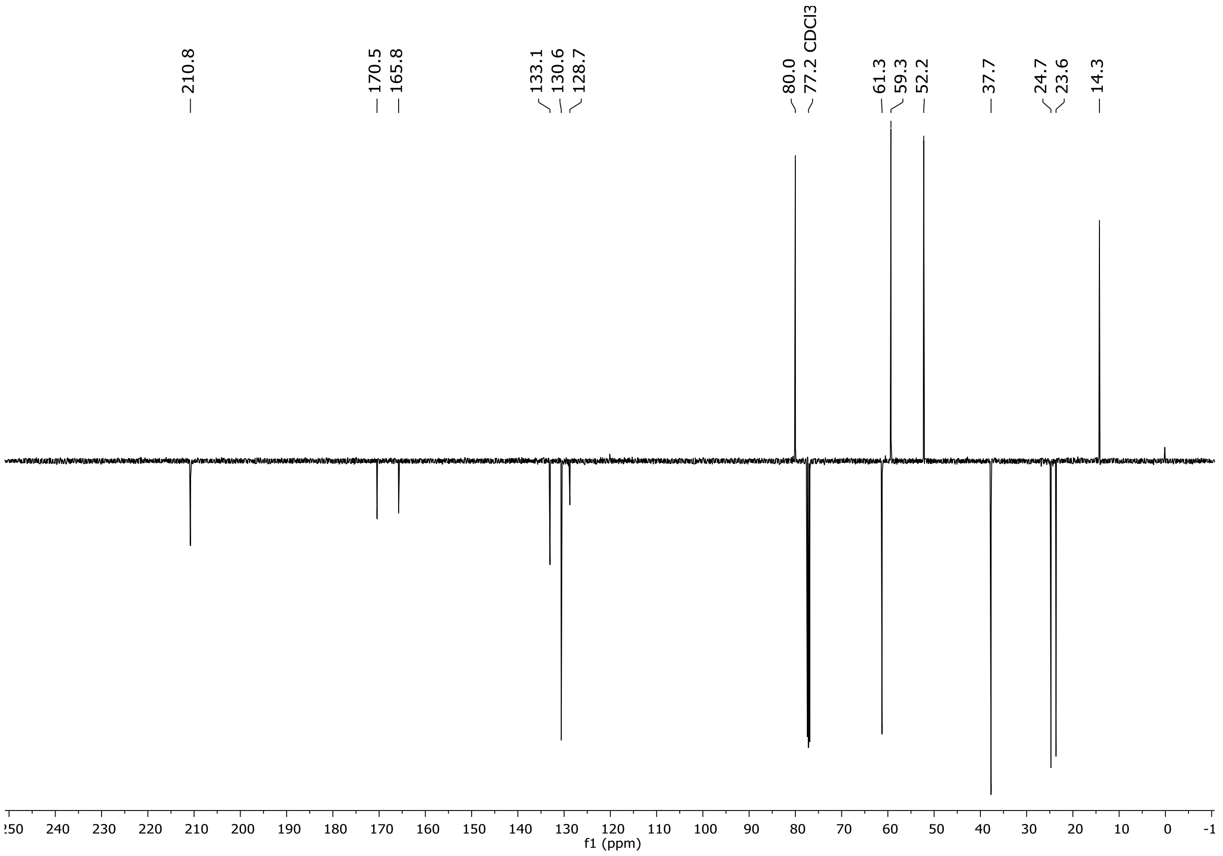

1.5.6 ^1H (500 MHz, $CDCl_3$) and ^{13}C (125 MHz, $CDCl_3$) spectra of ItaCORM 2b

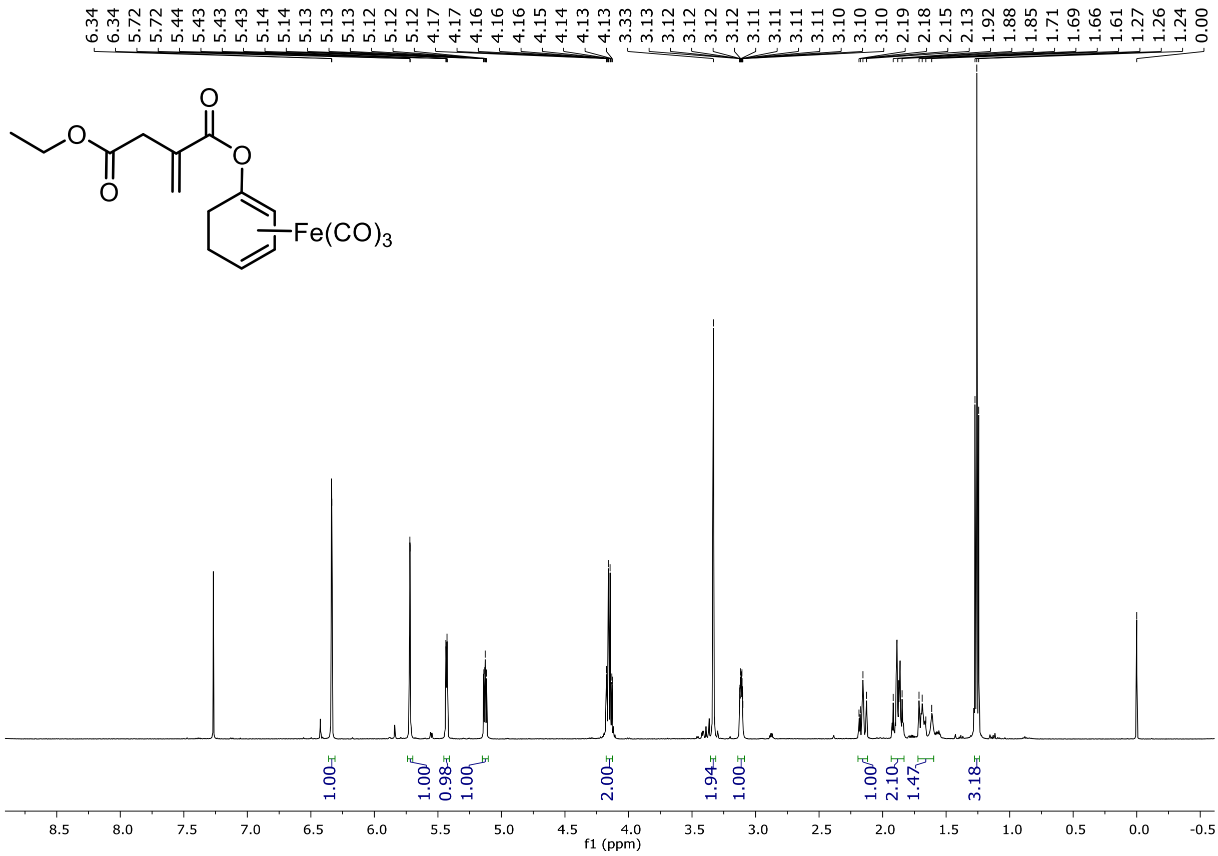

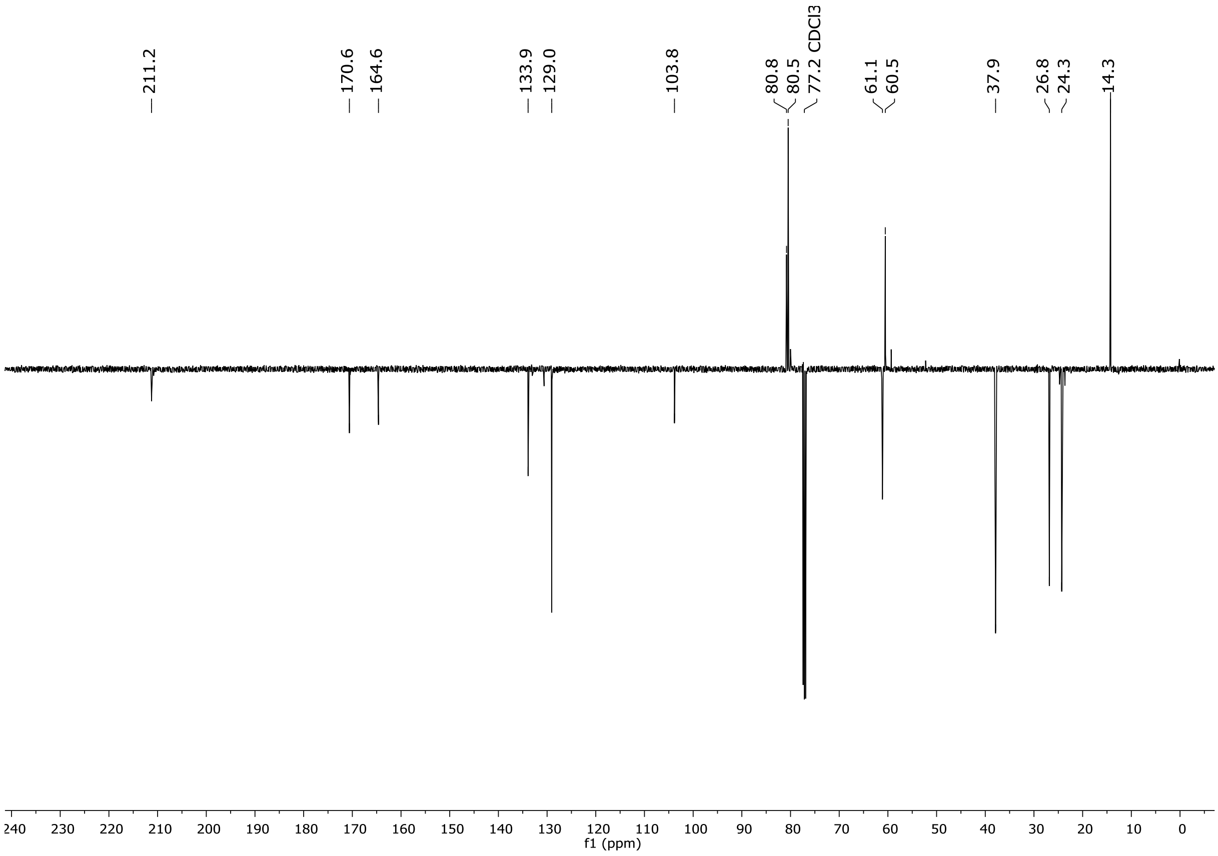

1.5.7 ^{1}H (500 MHz, $CDCl_3$) and ^{13}C (125 MHz, $CDCl_3$) spectra of ItaCORM 3a

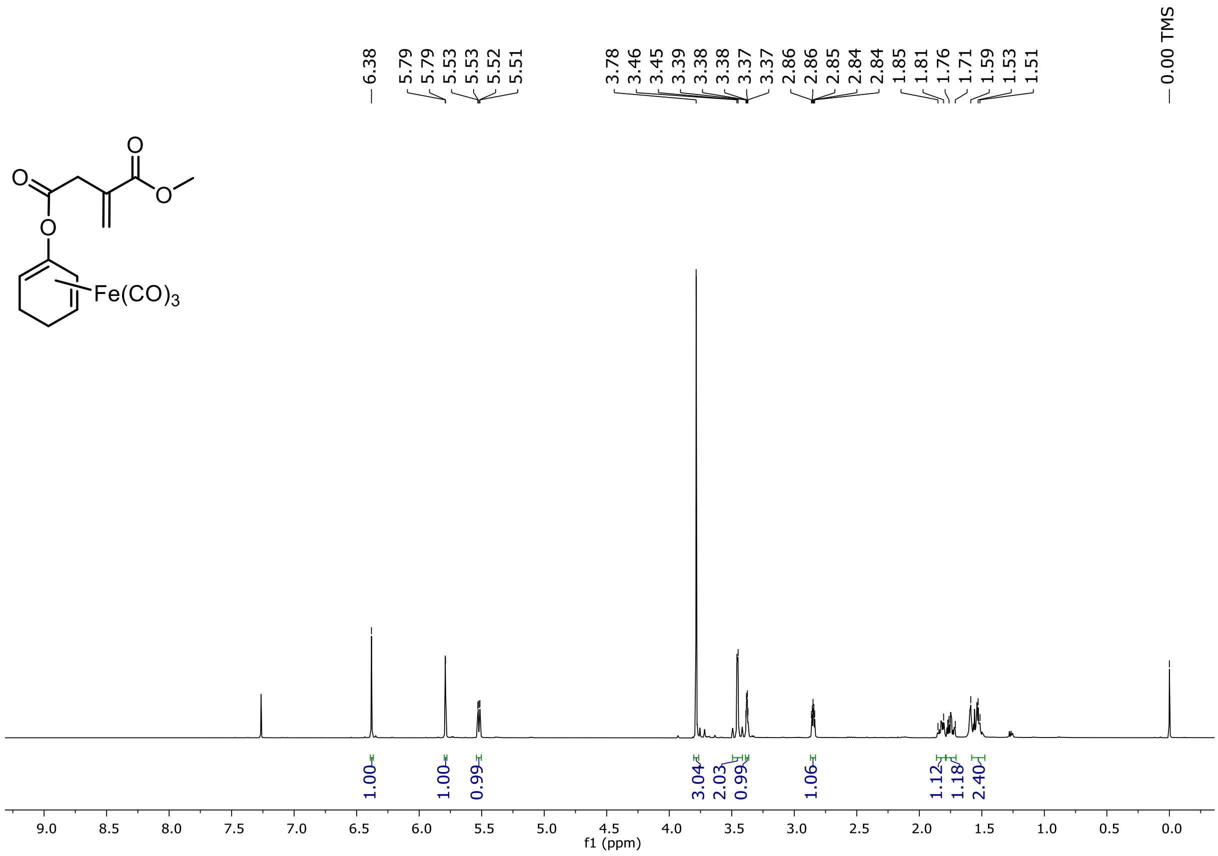

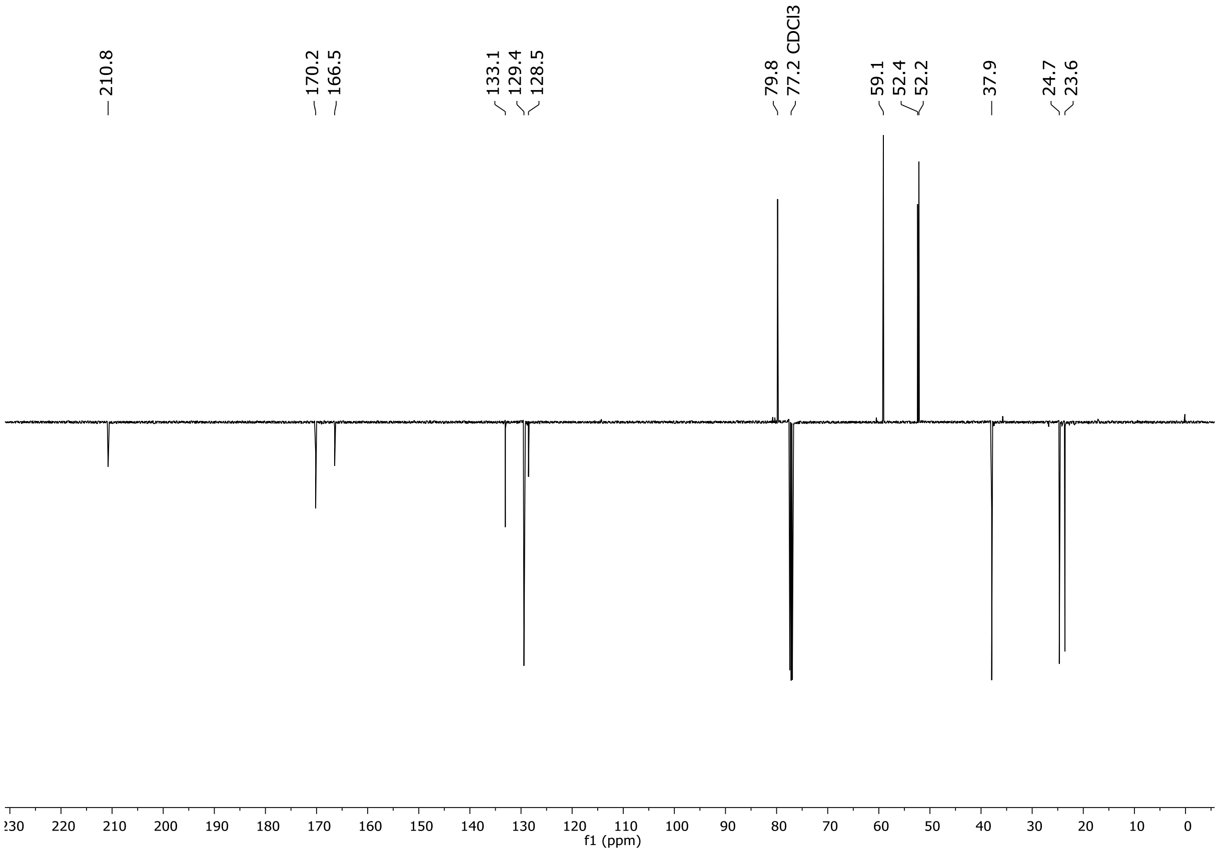

1.5.8 ^{1}H (500 MHz, $CDCl_3$) and ^{13}C (125 MHz, $CDCl_3$) spectra of ItaCORM 3b

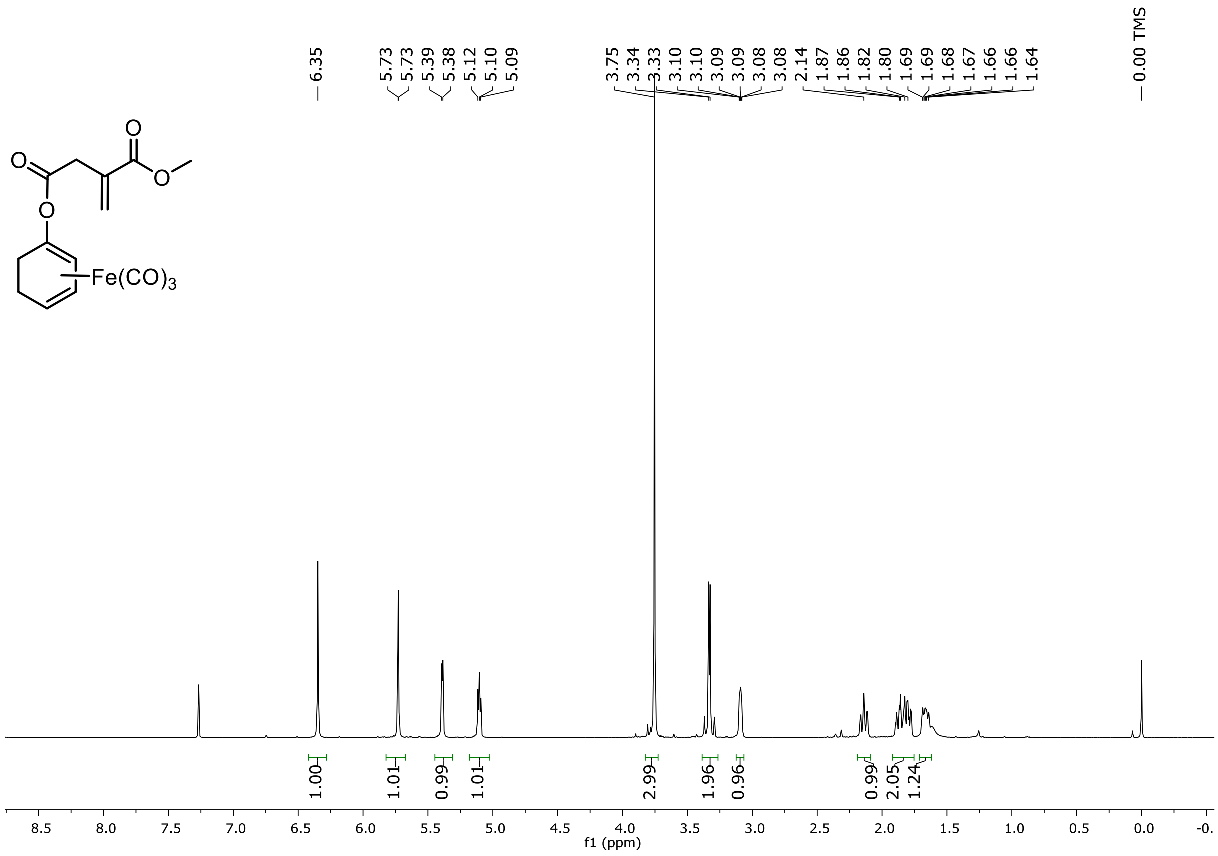

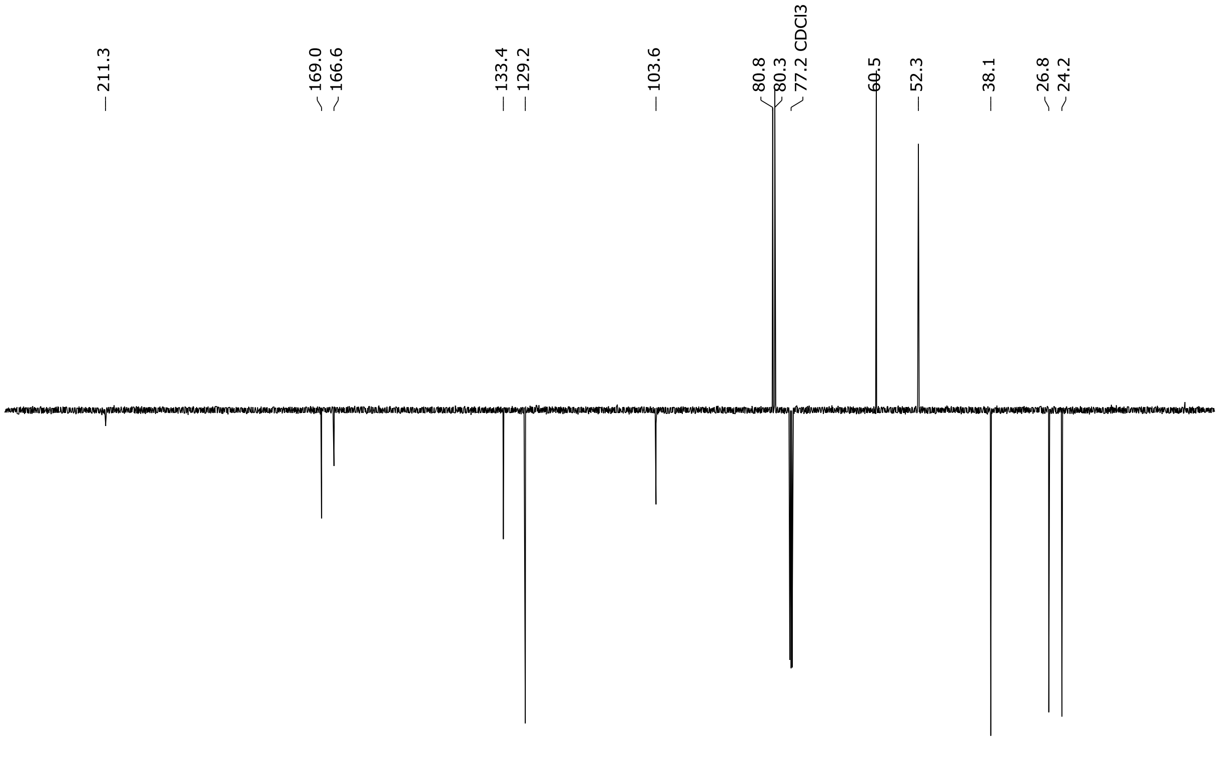

1.5.9 ^{1}H (500 MHz, $CDCl_3$) and ^{13}C (125 MHz, $CDCl_3$) spectra of ItaCORM 4a

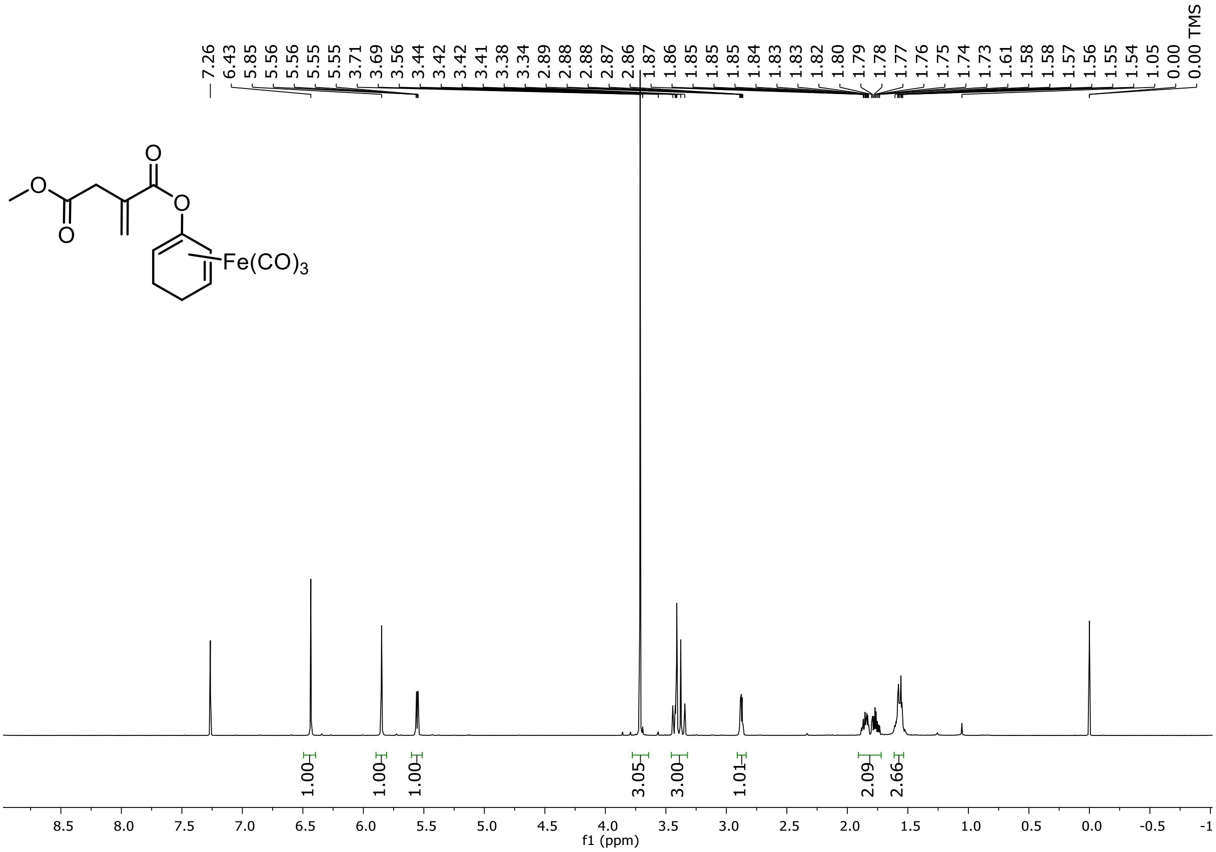

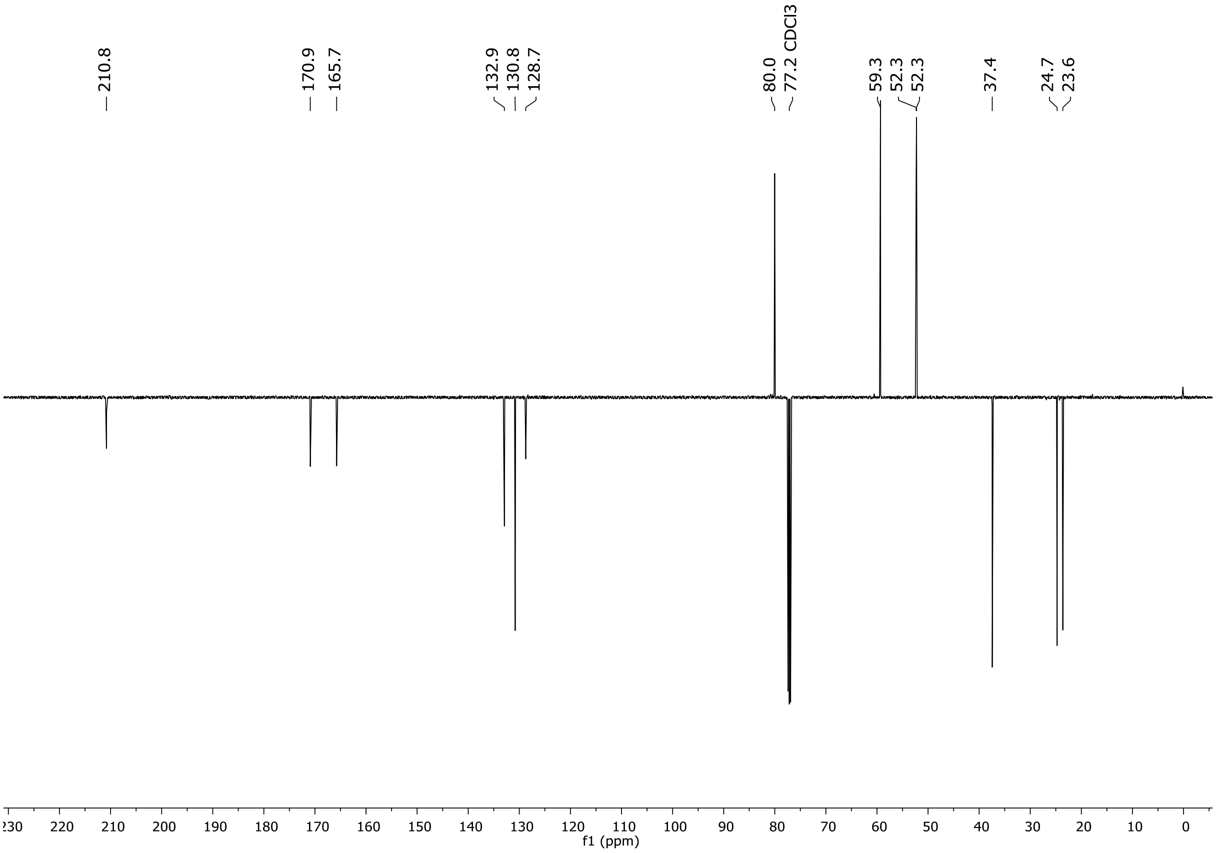

1.5.10 ^{1}H (500 MHz, $CDCl_3$) and ^{13}C (125 MHz, $CDCl_3$) spectra of ItaCORM 4b

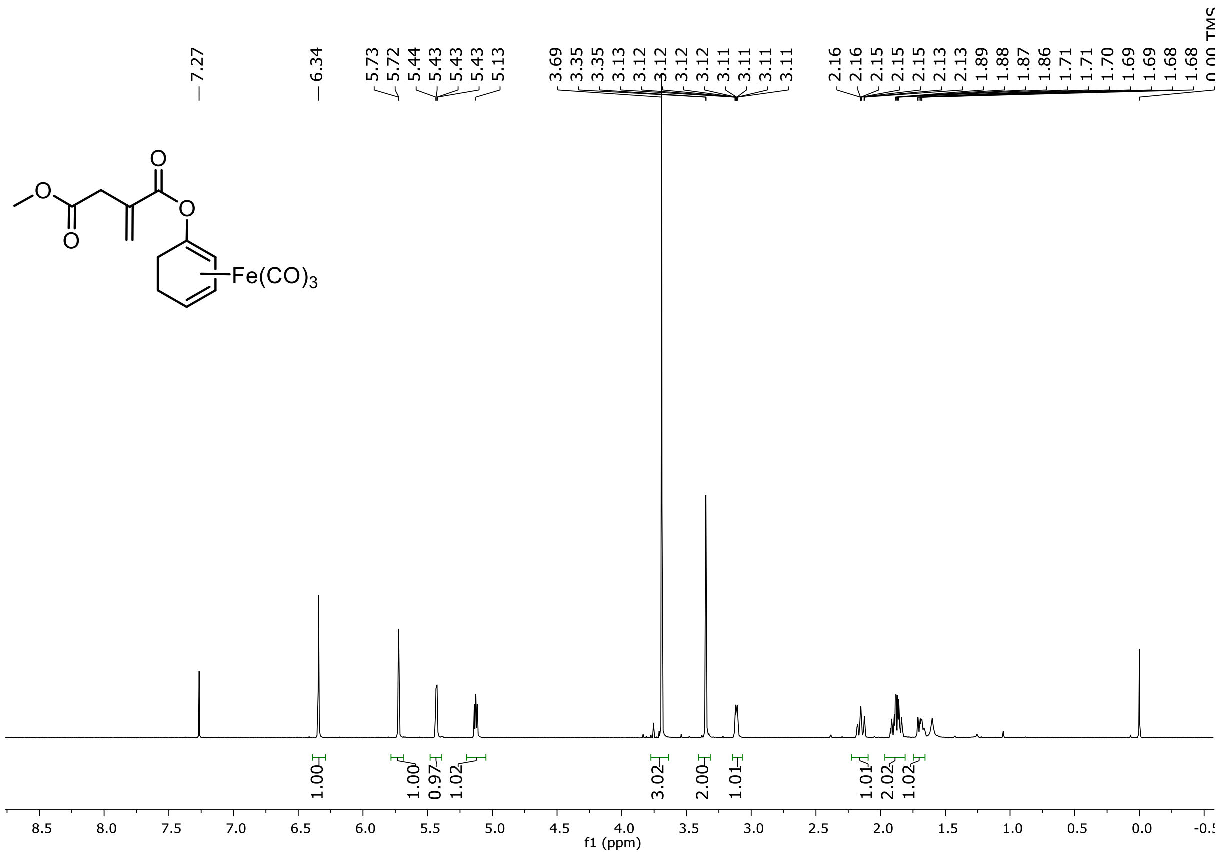

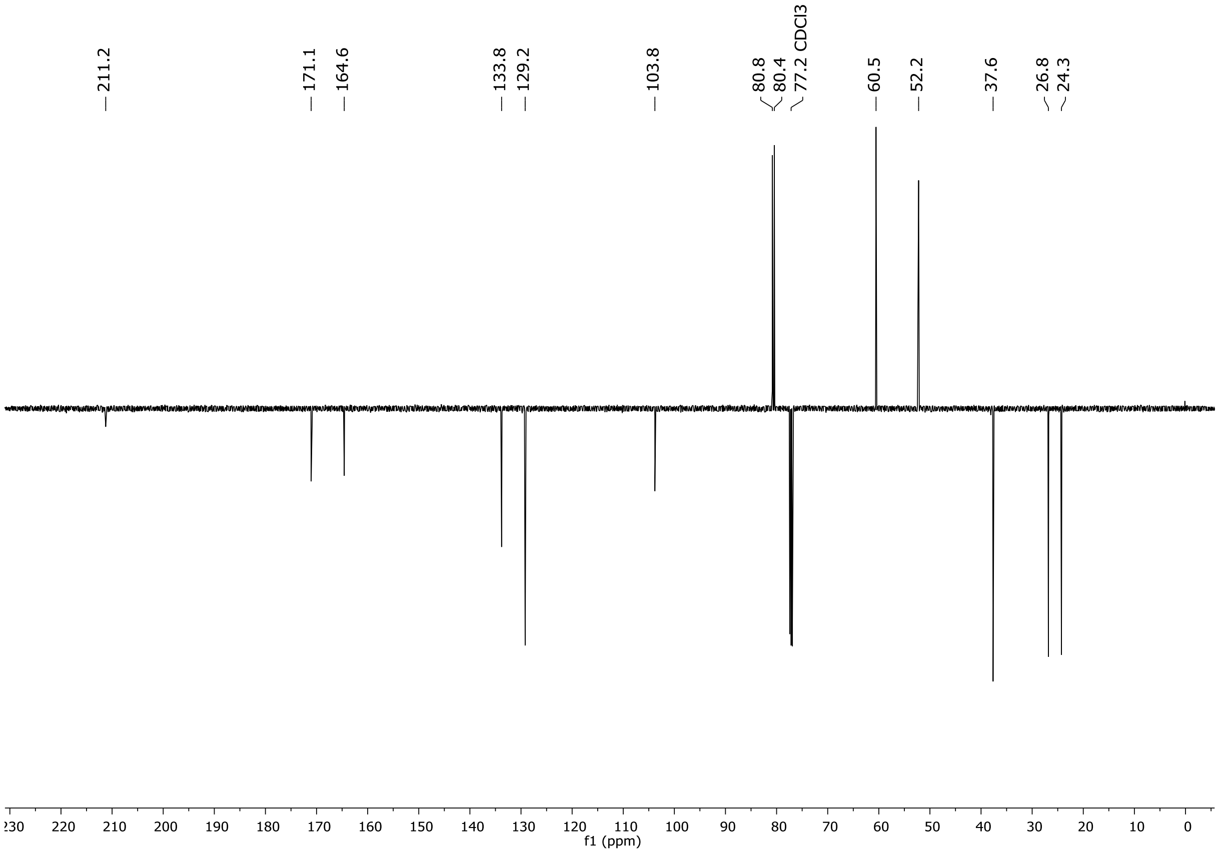

1.6 Crystal data and structure refinement

1.6.1 ItaCORM 3b

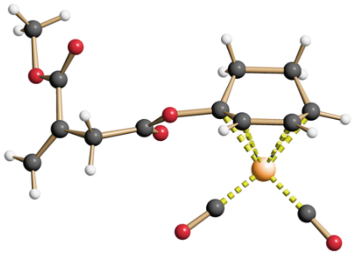

CCDC	2077461	
Identification code	bhd_kse9_nm (= ItaCORM **3b**)	
Empirical formula	C15 H14 Fe O7	
Moiety formula	C15 H14 Fe O7	
Formula weight	362.11	
Temperature	100(2) K	
Wavelength	1.54178 Å	
Crystal system	Monoclinic	
Space group	$P2_1$	
Unit cell dimensions	a = 7.1067(3) Å	a= 90°.
	b = 12.2043(5) Å	b= 104.110(2)°.
	c = 9.3734(4) Å	g = 90°.
Volume	788.45(6) Å^3	
Z	2	
Density (calculated)	1.525 Mg/m^3	
Absorption coefficient	7.987 mm^{-1}	
F(000)	372	
Crystal size	0.150 x 0.100 x 0.030 mm^3	
Theta range for data collection	4.865 to 71.892°.	
Index ranges	-8<=h<=8, -15<=k<=15, -11<=l<=11	
Reflections collected	14895	
Independent reflections	3057 [R(int) = 0.0603]	
Completeness to theta = 67.679°	99.9 %	
Absorption correction	Semi-empirical from equivalents	
Max. and min. transmission	0.7535 and 0.4517	
Refinement method	Full-matrix least-squares on F^2	
Data / restraints / parameters	3057 / 1 / 210	
Goodness-of-fit on F^2	1.071	
Final R indices [I>2sigma(I)]	R1 = 0.0431, wR2 = 0.1008	
R indices (all data)	R1 = 0.0471, wR2 = 0.1039	
Absolute structure parameter	0.467(8)	
Extinction coefficient	n/a	
Largest diff. peak and hole	1.104 and -0.293 e.Å^{-3}	

1.6.2 ItaCORM 4b

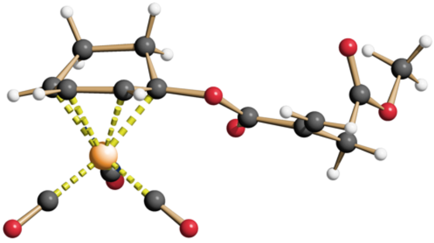

CCDC	2077462	
Identification code	bhd_kse10n (= ItaCORM **4b**)	
Empirical formula	C15 H14 Fe O7	
Moiety formula	C15 H14 Fe O7	
Formula weight	362.11	
Temperature	100(2) K	
Wavelength	1.54178 Å	
Crystal system	Orthorhombic	
Space group	Pbcn	
Unit cell dimensions	a = 11.3484(2) Å	a= 90°.
	b = 7.47810(10) Å	b= 90°.
	c = 36.7370(6) Å	g = 90°.
Volume	3117.67(9) $Å^3$	
Z	8	
Density (calculated)	1.543 Mg/m^3	
Absorption coefficient	8.079 mm^{-1}	
F(000)	1488	
Crystal size	0.150 x 0.150 x 0.030 mm^3	
Theta range for data collection	4.580 to 72.285°.	
Index ranges	-13<=h<=10, -9<=k<=9, -45<=l<=45	
Reflections collected	33111	
Independent reflections	3074 [R(int) = 0.0511]	
Completeness to theta = 67.679°	99.9 %	
Absorption correction	Semi-empirical from equivalents	
Max. and min. transmission	0.7536 and 0.3992	
Refinement method	Full-matrix least-squares on F^2	
Data / restraints / parameters	3074 / 0 / 209	
Goodness-of-fit on F^2	1.093	
Final R indices [I>2sigma(I)]	R1 = 0.0360, wR2 = 0.0935	
R indices (all data)	R1 = 0.0381, wR2 = 0.0945	
Extinction coefficient	n/a	
Largest diff. peak and hole	1.342 and -0.511 e.$Å^{-3}$	

2 Biology – Experimental Procedures

2.1 Animals

For the experiments performed, primary dendritic cells were isolated from C57BL/6 mice aged 8 weeks or older. The animals were bred and held in the facilities of the Eberhard Karls University Tübingen under specific pathogen free conditions. Mice were handled in accordance with the guidelines of the local authorities (Regierungspräsidium Tübingen, Germany).

2.2 Isolation of primary dendritic cells

The isolation of bone marrow derived dendritic cells (BMDCs) was performed according to a protocol of Madaan *et al.*.[4] Initially, the bone marrow was isolated from femurs and tibias of C57BL/6 mice. After erythrocyte lysis, the cell suspension was plated with 2x10^6 cells/ml on petri dishes (non-tissue culture grade). The culture medium consisted of DMEM supplemented with 10 % fetal calf serum, 1 % non-essential amino acids, 1 % sodium pyruvate, 1 % HEPES, 1 % antibiotics, 25 µM ß-mercaptoethanol and 20 ng/ml recombinant granulocyte-macrophage colony-stimulating factor (GM-CSF). On day 3 and day 6 after plating of the cells, replenishment of culture medium was conducted by addition of fresh medium. Immature dendritic cells were harvested on day 7 by careful collection of non-adherent cells. Finally, immature BMDCs were plated for subsequent *in vitro* experiments with 1x10^6 cells/ml.

2.3 Viability

BMDCs were treated with 1 µg/ml LPS for 24 h and increasing concentrations of **ItaCORM**s. Then, cell viability was determined using two different methods: (i) the Cell Proliferation Kit II (XTT) from Merck KGaA and (ii) the Pierce LDH Cytotoxicity Assay Kit from Thermo Fisher Scientific. Analyses were performed as described in the manufacturer's protocols.

2.4 Western blot

For the analyses of the HO-1 and STAT1 signaling pathways, BMDCs were stimulated with 1 µg/ml LPS and **ItaCORM**s or their corresponding reference substances for 2 h. Subsequently, Western blotting was performed as previously described in detail.[5] In the present study, α-tubulin (1:5000; Novus Biologicals) was applied as loading control.

2.5 Analyses of cytokines

For the analyses of inflammatory cytokine levels, BMDCs were treated with 1 µg/ml LPS and **ItaCORM**s or their respective reference substances. After 24 h of stimulation, the cell culture supernatant was collected. Measurements of IL-12p70 and IL-23 concentrations were performed using the commercially available kits DuoSet ELISA Mouse IL-12p70, DuoSet ELISA Mouse IL-23 in combination with the DuoSet Ancillary Reagent Kit 2 from R&D Systems. The whole procedure was performed as described in the manufacturer's protocol.

3 Biology - Results

3.1 Determination of cell viability

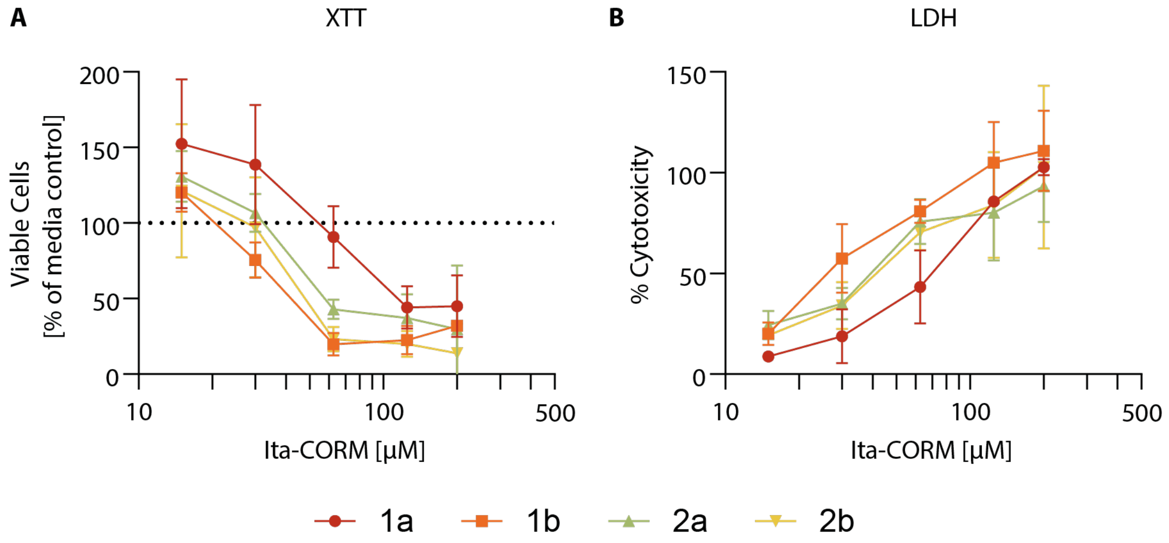

Figure 9: Analysis of cell viability after ItaCORM treatment.
BMDCs were treated with PBS, 1 µg/ml LPS and the indicated concentrations of **ItaCORM**s for 24 h. Cell viability was then measured using XTT (A) and LDH (B) assay. Shown is the percentage of viable cells (A) or cytotoxicity (B) in relation to the PBS-treated control. Values are means ± SEM (n=3).

3.2 ELISA

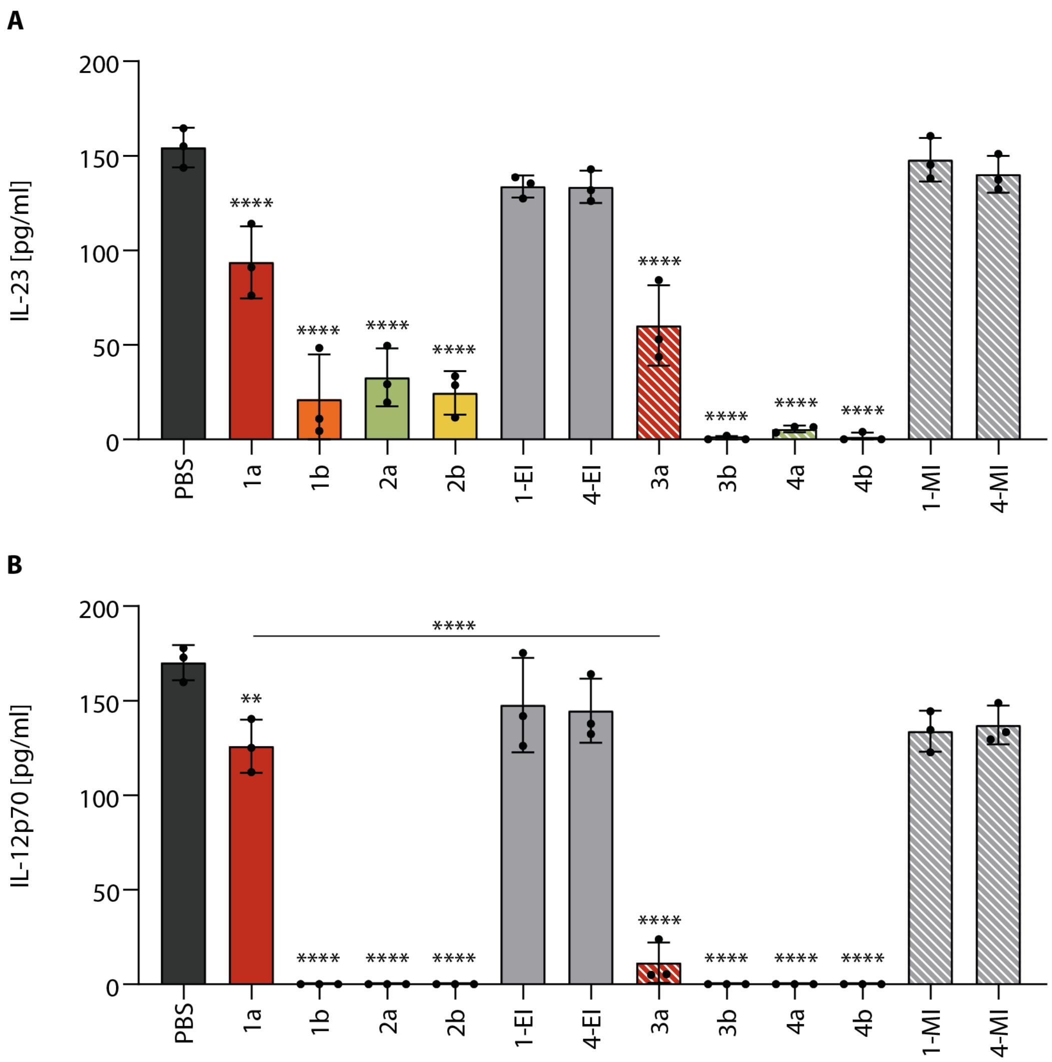

Figure 10: Inhibition of inflammatory cytokines.
BMDCs were stimulated with 1 µg/ml LPS and co-incubated with PBS, 15 µM **ItaCORM**s or the respective reference substances. After 24 h incubation, the cell culture supernatant was harvested and analyzed for IL-23 (**A**) and IL-12p70 (**B**) concentrations. Values are means ± SEM; **, $p \leq 0.01$; ****, $p \leq 0.0001$ (ANOVA with Tukey post-hoc test).

4 References

1. A. F. Burchat, J. M. Chong and N. Nielson, *Organomet. Chem.*, 1997, **542**, 281-283.
2. P. Ferraboschi, S. Casati, P. Grisenti and E. Santaniello, *Tetrahedron*, 1994, **50**, 3251-3258.
3. K. Achiwa, P. A. Chaloner and D. Parker, *Organomet. Chem.*, 1981, **218**, 249-260.
4. A. Madaan, R. Verma, A. T. Singh, S. K. Jain and M. Jaggi, *J. Biol. Methods*, 2014, **1**, e1.
5. B. Bauer, A.-L. Göderz, H. Braumüller, J. M. Neudörfl, M. Röcken, T. Wieder and H.-G. Schmalz, *Chem. Med. Chem.*, 2017, **12**, 1927-1930.
